# Boosting Heterologous Phenazine Production in *Pseudomonas putida* KT2440 Through the Exploration of the Natural Sequence Space

**DOI:** 10.3389/fmicb.2019.01990

**Published:** 2019-08-28

**Authors:** Theresia D. Askitosari, Santiago T. Boto, Lars M. Blank, Miriam A. Rosenbaum

**Affiliations:** ^1^Institute of Applied Microbiology – iAMB, Aachen Biology and Biotechnology – ABBt, RWTH Aachen University, Aachen, Germany; ^2^Leibniz Institute for Natural Product Research and Infection Biology – Hans Knöll Institute, Jena, Germany; ^3^Faculty of Biological Sciences, Friedrich Schiller University Jena, Jena, Germany

**Keywords:** *Pseudomonas putida*, phenazine, PCA, pyocyanin, heterologous production, bioelectrochemical systems

## Abstract

Phenazine-1-carboxylic acid (PCA) and its derivative pyocyanin (PYO) are natural redox mediators in bioelectrochemical systems and have the potential to enable new bioelectrochemical production strategies. The native producer *Pseudomonas aeruginosa* harbors two identically structured operons in its genome, which encode the enzymes responsible for PCA synthesis [*phzA1-G1* (operon 1), *phzA2-G2* (operon 2)]. To optimize heterologous phenazines production in the biotech host *Pseudomonas putida* KT2440, we compared PCA production from both operons originating from *P. aeruginosa* strain PAO1 (*O1.phz1* and *O1.phz2*) as well as from *P. aeruginosa* strain PA14 (*14.phz1* and *14.phz2*). Comparisons of phenazine synthesis and bioelectrochemical activity were performed between heterologous constructs with and without the combination with the genes *phzM* and *phzS* required to convert PCA to PYO. Despite a high amino acid homology of all enzymes of more than 97%, *P. putida* harboring *14.phz2* produced 4-times higher PCA concentrations (80 μg/mL), which resulted in 3-times higher current densities (12 μA/cm^2^) compared to *P. putida* 14.phz1. The respective PCA/PYO producer containing the *14.phz2* operon was the best strain with 80 μg/mL PCA, 11 μg/mL PYO, and 22 μA/cm^2^ current density. Tailoring phenazine production also resulted in improved oxygen-limited metabolic activity of the bacterium through enhanced anodic electron discharge. To elucidate the reason for this superior performance, a detailed structure comparison of the PCA-synthesizing proteins has been performed. The here presented characterization and optimization of these new strains will be useful to improve electroactivity in *P. putida* for oxygen-limited biocatalysis.

## Introduction

Bioelectrochemical systems or BES can be utilized to generate bioelectricity from renewable (often waste) carbon sources or to sustainably produce platform chemicals through the interaction of microorganisms with a polarized electrode (Bajracharya et al., 2016). As a bioprocessing system, BES involve a microbial whole-cell biocatalysts to drive oxidation and reduction (redox) reactions at solid electrodes (Rosenbaum and Henrich, 2014). The electron exchange can thereby be either performed through a direct electron transfer (DET) mechanism, involving a physical contact between the cell and the electrode and typically requiring specific redox proteins, or a mediated electron transfer (MET), involving a soluble redox shuttling compound as the main electron transfer pathway. These two fundamental electron transfer modes will have a strong strategic consequence for the development of future bioprocesses, enabled or enhanced through microbial electroactivity. From a biotechnological standpoint, connecting a solution-based MET process to a target microbial production hosts has the great advantage to be easily compatible with classical bioprocess technology of stirred tank reactors. The optimization goal then would be to insert electrodes into the bioreactor infrastructure at as low as possible disturbance of the reactor fluid dynamics (Krieg et al., 2019). In contrast, a DET process would demand for completely new reactor concepts since the biochemical production would be limited to a two dimensional biofilm and a maximum packing density of biofilm and electrode material would be required for the bioreactor to reach high titers and productivities.

For MET processes, the electron mediator can be exogenous (artificially added in the system) or endogenous (naturally synthesized by the microorganism) (Harnisch et al., 2011). Advantages of endogenous mediators over exogenous mediators, like methylene blue (Rahimnejad et al., 2011), neutral red (Popov et al., 2012) or ferricyanide (Hu et al., 2015; Lai et al., 2016), are that they might be more biocompatible, do not require costly addition of chemical compounds, are likely produced throughout the process, and the production is linked to the activity of the producing microorganism. A disadvantage is that only certain microbial species are naturally equipped to produce endogenous redox mediators for extracellular electron transfer.

One group of these redox compounds studied for natural MET are microbial phenazines, which are produced by *Pseudomonas* species, especially by *Pseudomonas aeruginosa*. Among other functions, phenazines like phenazine-1-carboxylic acid (PCA) and pyocyanin (PYO) play an important role in anaerobic survival of *P. aeruginosa* through discharging surplus metabolic electrons to not directly accessible terminal electron acceptors, such as far away oxygen, iron oxides, or an anode (Rabaey et al., 2005; Pierson and Pierson, 2010; Glasser et al., 2014). The complex regulatory network of phenazine synthesis and the pathogenic nature of *P. aeruginosa* limit the application of phenazine redox mediation with this host for specific electrobiotechnological applications (Venkataraman et al., 2011). Thus, to avoid *P. aeruginosa* as microbial host, the utilization of a non-pathogenic species for heterologous phenazines production would be an advantage for future biotechnological utilization. However, because of their redox activity, phenazines also have strong antimicrobial activity through the generation of reactive oxygen species for those microorganisms, which do not possess a strong defense system against oxygen radicals. For example the top biotech host *Escherichia coli* is unable to tolerate the reactive phenazine PYO. On the other hand, tolerant microorganisms can make energetic use of provided phenazines by using them as a vehicle to allow for an anaerobic respiration under anaerobic conditions as it has been shown for the natural habitat partner *Enterobacter aerogenes* (Venkataraman et al., 2011; Schmitz and Rosenbaum, 2018).

One non-pathogenic *Pseudomonas* species closely related to the native phenazine producer and tolerant to phenazines, which has emerged as a versatile recombinant biotech host, is *Pseudomonas putida* KT2440. This host stands out because of the availability of extensive genetic and intrinsic metabolism information, combined with its tolerance to many relatively unpolar or even toxic chemical substances. Therefore, it has become very useful as a production host of various valuable compounds (Loeschcke and Thies, 2015). However, one current drawback of *P. putida* as production host is its obligate aerobic metabolism, which cannot always be sustained even in well mixed stirred tank reactors. So far, attempts to engineer alternative respirative or fermentative pathways showed only limited success (Nikel and de Lorenzo, 2013; Steen et al., 2013).

In a successful initial proof-of-principle study, we introduced an engineered strain of *P. putida* KT2440, in which heterologous phenazine synthesis enabled electron discharge to an extracellular anode under oxygen-limited conditions in BES (Schmitz et al., 2015). The heterologous expression of the nine phenazine synthesis genes from *P. aeruginosa* PAO1 (*phzA1-G1, phzM*, and *phzS*) in *P. putida* KT2440 resulted in the production of PCA and PYO, whereby the derivative PYO was only fully synthesized when active aeration (AA) was applied during the initial growth phase since the final enzyme PhzS is dependent on molecular oxygen. During subsequent passive aeration (PA) via opened vent filter (i.e., strong oxygen limitation of the culture), metabolic activity sustained over 10 days and PCA was further synthesized and accumulated. Thereby, the total produced phenazine concentrations in BES were in the upper range of phenazine concentrations produced by the native producer *P. aeruginosa* [30–40 μg/mL for *P. putida* pPhz (Schmitz et al., 2015) vs. 22–25 μg/mL for *P. aeruginosa* PA14 (Bosire et al., 2016)].

In order to develop an efficient *P. putida* biocatalyst for oxygen-limited bioelectrochemical production, such as the production of rhamnolipids (Wittgens et al., 2011) or aromatics (Wierckx et al., 2005), it is necessary to further explore and optimize the heterologous production of phenazine. The work conducted by Bosire et al. (2016) showed for several carbon sources that *P. aeruginosa* PA14 produced more phenazines and higher resulting anodic currents than *P. aeruginosa* PAO1 in oxygen-limited BES. This indicated that the phenazine production in *P. aeruginosa* is strain dependent.

Furthermore, *P. aeruginosa* encodes for two homologous operons; *phzA1-G1* (operon 1) and *phzA2-G2* (operon 2), which are responsible for the synthesis of PCA under different regulatory conditions. Although the two phenazine operons of *P. aeruginosa* are 98% identical on DNA level (97% in amino acid level), they differ distinctively in their promoter regions. A *las* box is present in the promoter region of the *phz1* operon 390 bp upstream of the *phzA1* translational region. With this regulatory genetic element, the quorum sensing regulators LasR and/or RhlR recognize their target genes to initiate quorum sensing controlled gene expression. In contrast, for *phzA2*, the *las* box is not present in the promoter region (Recinos et al., 2012). A more detailed analysis by Mavrodi et al. (2001) revealed that the two operons contain most dissimilarities at DNA sequence level within *phzA1B1* and *phzA2B2*, while the regions from *phzC* to *phzG* of both operons are highly identical (Mavrodi et al., 2001). Hence, the heterologous expression of those two operons might be markedly different.

Therefore, in this study, we generated new strains of *P. putida* expressing phenazine genes from three different gene sources as follows; *P. aeruginosa* PAO1 (operon 1) and *P. aeruginosa* PA14 (operons 1 and 2) to produce PCA. Those three new constructs were compared with the performance of the already existing constructs originating from *P. aeruginosa* PAO1 operon 1 (Schmitz et al., 2015). The PCA producing- *P. putida* strains were combined further with the *phzM*+*S* plasmid to produce PYO. All phenazine producer strains were characterized for two different oxygen-limited conditions in BES; (i) 48 h active aeration (AA) during initial growth then subsequently switched to passive headspace aeration, and (ii) fully passive aeration (PA) throughout the experiment. With this approach, we aim to obtain the best heterologous *P. putida* phenazines producer for efficient biocatalysis under oxygen-limited condition BES.

## Materials and Methods

### Bacterial Strains, Media Preparation, and Strain Cultivation

*Pseudomonas putida* strain KT2440 (DSM 6125) was used for heterologous expression of phenazine genes from *P. aeruginosa* PAO1 (DSMZ 19880) and PA14 (DSMZ 19882). *Escherichia coli* strain DH5α (New England Biolabs) has been used as intermediary cloning recipient. Standard strain cultivations were performed in 250 mL shake flasks using LB medium (Carl Roth^®^) with or without antibiotics as required and were incubated at 30°C (*P. putida*) or 37°C (*E. coli*). For *P. putida* characterization in well plates, shake flasks and BES experiment, the strains have been cultivated at 30°C in Delft mineral salt medium (Hartmans et al., 1989), with a final composition (per L) of 3.88 g K_2_HPO_4_ (22 mM), 1.63 g NaH_2_PO_4_ (14 mM), 2.00 g (NH_4_)_2_SO_4_, 0.1 g MgCl_2_.6H_2_O, 10 mg EDTA, 2 mg ZnSO_4_.7H_2_O, 1 mg CaCl_2_.2H_2_O, 5 mg FeSO_4_.7H_2_O, 0.2 mg Na_2_MoO_4_.2H_2_O, 0.2 mg CuSO_4_.5H_2_O, 0.4 mg CoCl_2_.6H_2_O, 1 mg MnCl_2_.2H_2_O, with 20 mM glucose (PA) or 40 mM glucose (AA) and antibiotics as required (kanamycin at 50 μg/mL concentration or/and gentamycin at 30 μg/mL concentration).

### Genetic Engineering of *P. putida* for Phenazine Synthesis

Table 1 gives an overview on the strains and plasmids used for construct generation. The pBNT plasmid harboring *phzA1-G1* amplified from *P. aeruginosa* PAO1 genomic DNA (*O1.phz1*) was generated by Schmitz et al. (2015). The other three plasmids to synthesize PCA were newly constructed in this study; plasmid pBNT.O1.phz2 harboring *phzA2-G2* amplified from *P. aeruginosa* PAO1; the plasmid pBNT14.phz1 and pBNT14.phz2 harboring *phzA1-G1* and *phzA2-G2*, respectively, amplified from *P. aeruginosa* PA14. For plasmids assembly, the Gibson assembly method was used and performed according to the manufacturer’s instruction (New England Biolabs-Gibson Assembly) (Gibson et al., 2009).

**TABLE 1:** Bacterial strains and plasmids used for genetic engineering of *P. putida* for phenazine synthesis.

**Strains/Plasmids**	**Characteristics**	**Source**
**Strains**		
*P. aeruginosa* PAO1	Wildtype	DSMZ
*P. aeruginosa* PA14	Wildtype	DSMZ
*P. putida* KT2440	Wildtype	DSMZ
*P. putida* O1.phz1	Harboring plasmid pBNT.O1.phz1	Schmitz et al., 2015
*P. putida* O1.phz2	Harboring plasmid pBNT.O1.phz2	This study
*P. putida* 14.phz1	Harboring plasmid pBNT.14.phz1	This study
*P. putida* 14.phz2	Harboring plasmid pBNT.14.phz2	This study
*P. putida* O1.phz1+	Harboring plasmid pBNT.O1.phz1 and pJNN.phzM+S	Schmitz et al., 2015
*P. putida* O1.phz2+	Harboring plasmid pBNT.O1.phz2 and pJNN.phzM+S	This study
*P. putida* 14.phz1+	Harboring plasmid pBNT.14.phz1 and pJNN.phzM+S	This study
*P. putida* 14.phz2+	Harboring plasmid pBNT.14.phz2 and pJNN.phzM+S	This study
*E. coli* DH5α	*fhuA2 (argF-lacZ)U169 phoA glnV44 80 (lacZ)M15 gyrA96 recA1 relA1 endA1 thi-1 hsdR17*	NEB
**Plasmids**		
pBNT	ORI: ori/IHF for replication in *E. coli* and *Pseudomonas*; kanamycin resistance-cassette, salicylate-inducible *nagR*/*pNagAa* promoter	TU Delft, Netherlands
pJNN	ori RO1600 for *Pseudomonas* and ori ColE1 for *E.coli*; gentamycin resistance-cassette, salicylate-inducible *nagR*/*pNagAa* promoter	TU Delft, Netherlands
pBNT.O1.phz1	ORI: ori/IHF for replication in *E. coli* and *Pseudomonas*; kanamycin resistance-cassette, salicylate-inducible *nagR*/*pNagAa* promoter, *phzA1-G1.*PAO1 genes	Schmitz et al., 2015
pJNN.phzM+S	ori RO1600 for *Pseudomonas* and ori ColE1 for *E.coli*; gentamycin resistance-cassette, salicylate-inducible *nagR*/*pNagAa* promoter, *phzM* + *S.*PAO1 genes	Schmitz et al., 2015
pBNT.O1.phz2	ORI: ori/IHF for replication in *E. coli* and *Pseudomonas*; kanamycin resistance-cassette, salicylate-inducible *nagR*/*pNagAa* promoter, *phzA2-G2.* PAO1 genes	This study
pBNT.14.phz1	ORI: ori/IHF for replication in *E. coli* and *Pseudomonas*; kanamycin resistance-cassette, salicylate-inducible *nagR*/*pNagAa* promoter, *phzA1-G1.* PA14 genes	This study
pBNT.14.phz2	ORI: ori/IHF for replication in *E. coli* and *Pseudomonas*; kanamycin resistance-cassette, salicylate-inducible *nagR*/*pNagAa* promoter, *phzA2-G2.* PA14 genes	This study

Subsequently, all constructed plasmids have been transformed into *P. putida* KT2440 via electroporation (Choi et al., 2006). For combined PCA and PYO synthesis, the best pre-selected strain producing PCA from each operon origin was transformed further with pJNN.phzM+S generated by Schmitz et al. (2015). Transformed cells were selected on LB agar plates with kanamycin (K, 50 μg/mL) for PCA, while for PCA and PYO producers LB agar plates with kanamycin (same concentration) and gentamycin (G, 30 μg/mL) were used. The constructs were verified using colony PCR, restriction digest analysis and DNA sequencing with targeted primers (Supplementary Table S1). DNA plasmid sequencing analysis was performed using the Sanger sequencing method (GATC-Eurofins Genomics). The DNA sequencing products of the promoter and phenazine gene regions were compared and verified with the reference plasmid map using Clone Manager Software. The successful expression of the *phzA-G* genes will show the PCA production by exhibiting yellow color in Delft medium, while in combination with *phzM*+*S* genes, the bacterial culture produced PCA and PYO and appears in a greenish blue color.

### Aerobic Strain Characterization and Evaluation

Eight clones of each constructed strain were tested in triplicates for phenazine production in the multiplexed cultivation platform growth profiler (CR1424d, EnzyScreen, Heemstede, Netherlands), using 24 square-well plates filled with 5 mL Delft medium, 20 mM glucose, and supplemented with antibiotic/s (K, 50 μg/mL or/and G, 30 μg/mL). While the aerobic flask experiments were performed in 250 mL flasks; filled with 25 mL Delft medium. Both of the cultivations were starting at a uniform inoculation OD_600_: 0.1. After 3 h of incubation, sodium salicylate was added as an inducer of the phenazine gene expression. The incubation temperature was 30°C and the shaking frequency was 224 rpm (multiplexed cultivation) and 200 rpm (flasks experiment). The clone with the best growth as well as phenazine production obtained from each constructed strain was selected to be characterized further in the aerobic flasks as well as BES experiments.

### Oxygen-Limited Bioelectrochemical System Experiments

Single chamber glass BES reactors (500 mL working volume) equipped with a water jacket for the temperature control was used (Supplementary Figure S1). The integrated three electrode set-up included: a 156.32 cm^2^ graphite comb of high-grade graphite (EDM-3, Novotec) as working electrode (anode), a 49.22 cm^2^ graphite block as counter electrode (cathode), and an Ag/AgCl, saturated KCl reference electrode (192 mV vs. SHE at 30°C, pH 7) as reference electrode. The BES reactor experiments were performed at 30°C at 200 rpm stirring with a magnetic stirrer, and potentiostatically controlled by a potentiostat (VMP3, Biologic) at 0.2 V vs. reference electrode. The electrical current signal was recorded continuously including 24 h of blank media measurement. Sampling for measurements of optical density at 600 nm, pH and HPLC analysis were performed daily after the bacterial culture inoculation. The duplicate BES reactor experiments were performed under two types of oxygen-limited condition: AA, in which the active aeration was applied via a sparger at 30 mL/min flow rate for 48 h aeration followed by PA of the headspace via two opened vent filters, and PA, in which only passive aeration via opened vent filters was applied for the entire experiment.

### Phenazine Analysis

The quantification of phenazines was performed using high-performance liquid chromatography (HPLC) with a C_18_ column (Waters Corporation) equipped with a photodiode array UV/VIS detector (LC-168, Beckmann), which detected and quantified the PCA at 366 nm and PYO at 280 nm. Separation was achieved with a gradient of 0.1% trifluoroacetic acid (TFA) in acetonitrile (solution A) and 0.1% TFA in water (solution B) as mobile phase (with solution A at 15% for 2 min, 100% for 15 min, and 5% for 3 min) at a flow rate of 0.8 mL/min and a column temperature of 20°C (Kern and Newman, 2014). Analytical grade PCA (Princeton BioMolecular) and PYO (Cayman Chemical) were used as standard solutions of phenazines.

### Analysis of Sugar Metabolites

Analysis of glucose and secreted metabolites was performed using HPLC equipped with a 300 × 8.0 mm polystyroldivinylbenzol copolymer separation column (CS-Chromatographie), a UV/VIS detector at 210 nm and a refractive index (RI) detector at 35°C. The separation was achieved at 60°C with 5 mM sulphuric acid mobile phase at 0.8 mL/min isocratic flow rate. Analytical grade glucose, 2-ketogluconate, gluconate, and acetate (Carl Roth^®^) were used as standard solutions.

### Amino Acid Sequence Comparison of the Phenazine Operons

Sequences and annotations of *P. aeruginosa* PAO1 (assembly: GCA_000006765.1) and *P. aeruginosa* PA14 (assembly: GCA_000014625.1) were obtained from GenBank (Benson et al., 2005). Each strain contains two main clusters of seven genes organized in an operon for PCA production. All these genes were compared individually by multiple protein alignment and the entire operons (excluding the promoter) were compared by multiple nucleotide alignment. Clustal Omega 1.2.4 was used to perform the alignments (Sievers et al., 2011; Sievers and Higgins, 2018). In order to detect changes between the codon usage among genes in the four operons, codon adaptation indices/CAI (Sharp and Li, 1987) were calculated for all genes using an index previously generated for *P. putida* KT2440 (assembly: GCA_000007565.2). Plots for CAI comparison and operon analysis were created using RStudio (R Studio Team, 2015). All calculations were performed using Biopython 1.72 (Cock et al., 2009). For all proteins involved in phenazines production (except PhzF and PhzM due to full sequence identity), a 3D-model has been created by homology modeling using modeler (Webb and Sali, 2014). All structures were predicted in the same way, including those with an experimentally determined structure, to avoid bias. Models were built as homodimers, except PhzS that was built as a monomer. The predicted protein structures were superimposed and displayed using UCSF Chimera (Pettersen et al., 2004). RMSD (root-mean-square deviation of atomic positions) was calculated to measure the differences between structures.

## Results and Discussion

### Phenazine Gene Origin Determines the Phenazine Synthesis Capacity in *P. putida*

In an initial study, we transferred the capacity to synthesize the phenazines PCA and PYO from the pathogen *P. aeruginosa* into the GRAS (“generally recognized as safe”) organism *P. putida* KT2440 (Schmitz et al., 2015). The gene origins in that work came from *P. aeruginosa* strain PAO1 (i.e., *O1.phz1* and *phzM*+*S*). To test the hypothesis that the genetic origin of the encoding genes impacts phenazine synthesis, the two strongly homologous copies of the 7-gene operon responsible for PCA synthesis from two *P. aeruginosa* strains (*O1.phz1* and *O1.phz2* vs. *14.phz1* and *14.phz2*) were inserted into the inducible plasmid pBNT without their native promoter regions. Since the PhzM protein shares 100% amino acid identity, while the PhzS protein shares 99% amino acid identity in the two strains (without impact on the active site of the protein) (Supplementary Figure S13), the original plasmid construct for PYO synthesis (pJNN.phzM+S) with the genes originating from strain PAO1 was not changed in this study.

A comparison of phenazine synthesis of a strain version carrying only the PCA-production genes and the combination with the *phzM*+*S* genes to also synthesize PYO was undertaken (indicated by “ + ” right after the construct name, e.g., *P. putida* O1.phz1 makes only PCA and *P. putida* O1.phz1 + makes PCA and PYO). For each construct, eight colonies were picked from agar plates after transformation and compared in parallelized growth experiments on a micro-cultivation platform regarding the growth and yellow (PCA) or blue (PYO) pigment production (Supplementary Figures S2, S3).

**FIGURE 1:**
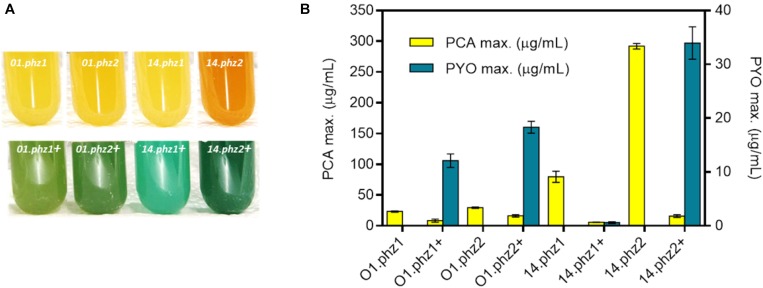
Heterologous expression of *phz* genes in *P. putida* KT2440. **(A)** *P. putida* cultures (from left to right) expressing O1.phz1, O1.phz2, 14.phz1, and 14.phz2 gene for PCA synthesis (top) and with combination of *phzM*+*S* genes for PCA and PYO synthesis PCA and PYO (bottom). **(B)** Phenazine production of engineered *P. putida* strains in triplicate shake flask experiments (fully aerobic), whereby the “+” in the strain designation indicates presence of *phzM*+*S* to convert PCA to PYO.

For a more quantitative evaluation of phenazine synthesis, the best clone of each construct in the micro-cultivations was further cultivated in fully aerobic shake flask experiments (Supplementary Figures S4, S5). The strain constructs *P. putida* 14.phz2 and *P. putida* 14.phz2+ showed the most intense color formation compared to other strains (Figure 1A) with the phenazine quantification results shown in Figure 1B. The *P. putida* O1.phz1 strain was found to be the lowest PCA producer (23.34 ± 1.06 μg/mL) and *P. putida* 14.phz1+ was identified to be the lowest PCA and PYO producer (5.86 ± 0.30 μg/mL PCA and 0.60 ± 0.15 μg/mL PYO, respectively). In comparison to the *P. putida* O1.phz1 strain, the expression of the 14.phz1 operon alone (without *phzM*+*S*) boosted PCA synthesis to 79.79 ± 9.09 μg/mL. Thus, PCA production of *P. putida* expressing the 14.phz1 operon was higher than with the O1.phz1 operon. However, the combination of both plasmids in *P. putida* to produce PCA and PYO worked better with *P. putida* O1.phz1 + than with *P. putida* 14.phz1 + where very little of both phenazines was produced (Figure 1B), although the growth curve of *P. putida* 14.phz1 + was similar compared to *P. putida* O1.phz1 + (Supplementary Figure S5). In *P. putida* 14phz.1+, the presence of the *phzM*+*S* genes obviously negatively impacted the PCA synthesis, which resulted in low PYO production.

By far the best PCA producer was *P. putida* 14.phz2 (292 ± 4.58 μg/mL) and *P. putida* 14.phz2+ was the strongest PCA and PYO producer (16 ± 2.31 and 33.96 ± 3 μg/mL, respectively). The growth curve of *P. putida* 14.phz2 was similar with *P. putida* O1.phz1 and *P. putida* 14.phz1. Thus, the *phzA-G* operon 2 from *P. aeruginosa* PA14 allowed higher PCA production compared to operon 1. In *P. putida* 14.phz2+, although PYO production was highest compared to the other strains, PCA production was low. This was surprising given the high PCA production of *P. putida* 14.phz2. However, the two plasmid strategy requires two antibiotics that might add up to a significant metabolic burden, causing the low phenazines synthesis. But it is also possible that the presence of PYO has a toxic effect on the cells, reduces the biosynthetic activity, and thereby hinders further PCA synthesis, since it is known that PYO generates toxic oxygen radicals.

Overall, the experiments conducted under fully aerobic conditions showed that the PCA synthesis of *P. putida* expressing the phenazine genes (same strain origin) from operon 2 of *P. aeruginosa* was higher than from operon 1. Furthermore, PCA production of *P. putida* expressing the phenazine genes from *P. aeruginosa* PA14 was higher compared to PAO1, while for combined PCA and PYO production, the phenazine production was affected by either the metabolic burden of the two antibiotics applied and/or a restricted total phenazine production with increased levels of PYO, which shows toxic behavior at higher concentration through the formation of reactive oxygen species (Mentel et al., 2009). In general, we can conclude that phenazine synthesis in *P. putida* is strain origin- and operon-dependent under fully aerobic condition.

### Phenazine Gene Origin Determines Electron Discharge Capacity in BES

**FIGURE 2:**
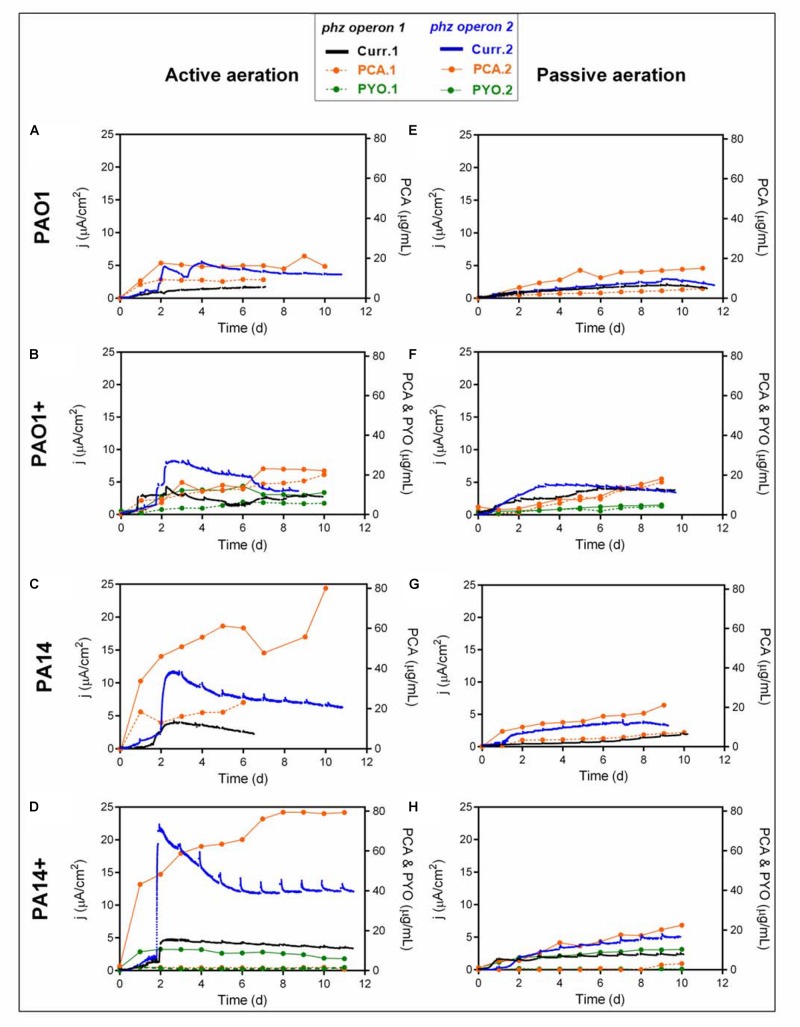
Heterologous phenazine production with *P. putida* under oxygen-limited conditions in a BES. Current densities generation and phenazine production of *P. putida* KT2440 harboring either *phz* operon 1 or operon 2 originating from *P. aeruginosa* strains PAO1 **(A,E,B,F)** or PA14 **(C,G,D,H)** under active **(A–D)** and passive **(E–H)** aeration. **(B,F,D,H)** indicated as PAO1+ and PA14+ are in combination with the *phzM*+*S* genes to synthesize both PCA and PYO. In the active aeration experiments, liquid phase aeration for the first 48 h was followed by passive headspace aeration. The highest currents generation and phenazine production were obtained from the engineered *P. putida* carrying operon *14.phz2*+ under active aeration BES (Figure 2D). The BES data are means from biological duplicates.

The motivation for engineering *P. putida* to produce phenazines is driven by the idea to capacitate this obligate aerobic bacterium for metabolic activity and in future for biocatalysis in oxygen-limited bioreactors (Schmitz et al., 2015). *P. putida* will be able to utilize phenazines as reversible redox mediators for the anodic discharge of metabolic electrons. We have shown with the native producer *P. aeruginosa* that the effectivity of anodic electron discharge with the two produced phenazines PCA and PYO can be fine-tuned by electrochemical potential control (Bosire and Rosenbaum, 2017). In future, this oxygen-limited metabolism will be coupled with biotechnological productions in the BES, such as the production of rhamnolipids, which currently suffers from technical challenges because of extensive foam formation during high level aeration (Beuker et al., 2016). In order to characterize the capacity of the different *P. putida* strains for electron discharge, the BES performance of each engineered strain variant has been evaluated with just initial aeration for the first 48 h (Figures 2A–D). In accordance with the shake flask experiments, the lowest PCA production and current generation were observed from *P. putida* O1.phz1, which only produced 9.38 μg/mL PCA, corresponding to 1.68 μA/cm^2^ current density (Figure 2A). A control experiment, where ∼17 μg/mL synthetic PCA was added to the *P. putida* KT2440 wildtype in an oxygen-limited BES, confirmed that the PCA concentration stayed constant in the reactor until the end of the experiment, i.e., was not degraded or absorbed to the electrode and a current density of 8.57 μA/cm^2^ was observed (Supplementary Figure S6A). Furthermore, by far the lowest PCA and PYO producer in actively aerated BES was *P. putida* 14.phz1+, which only produced 2.71 μg/mL PCA, 1.63 μg/mL PYO, and 4.74 μA/cm^2^ maximum current density (Figure 2D). This result also mirrored the very low activity of the phenazine production of *P. putida* 14.phz1+ in the flask experiments, where the combination of the *14.phz1* gene with the *phzM*+*S* genes led to an almost complete suspension of phenazine production.

The highest phenazine production and current generation was observed with *P. putida* 14.phz2, which produced 4-times higher PCA concentration, i.e., 80 μg/mL, resulting in 3-times higher maximum current density of 11.77 μA/cm^2^ compared to the other PCA gene clusters (Figure 2C). Also, the combination of PCA and PYO produced with *P. putida* 14.phz2+ outperformed the other strains with 80 μg/mL PCA, 11 μg/mL PYO, and 22.33 μA/cm^2^ maximum current density (Figure 2D). Again, the performance in oxygen-limited BES of this strain was in accordance with the aerobic flasks experiment. The presence of PYO as a soluble redox mediator in BES is markedly increasing electron shuttling to the anode. From control experiments with the *P. putida* KT2440 wildtype with ∼17 μg/mL exogenous PYO added to an oxygen-limited BES, we know that the PYO concentration is not sustained over time, likely because of adsorption to the electrode (Supplementary Figure S6B). Here, we see a fairly stable concentration of PYO over the course of the experiment with a continuously increasing concentration of PCA, suggesting continuous re-synthesis of PYO from PCA.

We also operated the BES under stringent oxygen limitation by only passively aerating the headspace (Figures 2E–H). Here, the lowest activities were observed for *P. putida* 14.phz1 (7.21 μg/mL of PCA, 2 μA/cm^2^ of current density) (Figure 2G) and *P. putida* 14.phz1 + (3.11 μg/mL of PCA, 0.65 μg/mL of PYO, 2.58 μA/cm^2^ current density) (Figure 2H). *P. putida* 14.phz2 was confirmed as the best PCA producer also under PA conditions with 21.09 μg/mL of PCA and 4.12 μA/cm^2^ current density (Figure 2G). Together with the *phzM*+*S* genes, *P. putida* 14.phz2+ was able to produce 22.48 μg/mL PCA and 10.29 μg/mL PYO, resulting in 5.53 μA/cm^2^ of current density (Figure 2H).

Overall, the performance of the *P. putida* phenazine producing strains in oxygen-limited BES is well in accordance with the performance in flasks experiment. Also, it is confirmed for these cultivation conditions that the phenazine synthesis in *P. putida* is strain origin- and operon-dependent. In direct comparison, the maximum produced amounts of PCA, however, are lower under oxygen-limited conditions compared to fully aerobic shake flasks because of the restricted energy availability. Notably, for the AA BES scenario, where the reactors were aerated for the first 48 h of growth to enable faster growth and cellular energy generation followed by completely PA with vent filters, a remarkable activity for PCA production resulting in roughly 1/3 of the concentrations of the aerobic cultivation was observed. This PCA production continued throughout the PA phase, confirming results from Schmitz et al. (2015) that stringently oxygen-limited conditions enable biosynthesis in *P. putida* if an anode is made accessible for metabolic electron discharge. The concentration of PYO did not further increase after switching from active to PA because of the requirement of molecular oxygen for PYO synthesis.

### Evaluation of Metabolic Effects of Phenazine Usage in BES

To further evaluate the metabolic activity of the phenazine producing *P. putida* strains under oxygen-limited conditions, we analyzed biomass formation, glucose consumption, metabolite production, and the efficiency of donating metabolic electrons derived from the substrate glucose to the anode, which is measured as coulombic efficiency. Table 2 summarizes all results. We did not observe any strong biofilm formation with *P. putida* in our BES, and the evaluations at OD_600_ are well suited to compare the growth performance of the strains. This is different from BES experiments with *P. aeruginosa* as described by Bosire et al. (2016), where we found very strong and variable biofilm formation in the experiments, making a time-resolved estimation of growth difficult. The best growth, with the highest OD_600_ of 2.45, was observed for *P. putida* 14.phz2+ under AA, which sustained throughout the stationary phase until day 11 after inoculation. The average OD_600_ for AA was 1.95 compared to 1.57 for PA, reflecting the very stringent oxygen availability during PA. The glucose consumption is shown as the percentage of the provided glucose, which were 40 mM in AA BES and 20 mM in PA BES. Most cultures showed a glucose consumption of around 80% for active and around 70% for PA. Notably, the lowest glucose consumption of only ∼40% for both active and PA was found for strain *P. putida* 14.phz1. It has been reported before that *P. putida* produces several organic acids, such as 2-ketogluconate, gluconate, and acetate as side products from glucose degradation under oxygen-limited conditions (Nikel et al., 2015; Schmitz et al., 2015). For most strains, side product formation in these experiments stayed fairly low in the range of 0.1–2.9 mM. However, the best phenazine and most active current producer *P. putida* 14.phz2+ reached up to 4.80 mM gluconate. This finding is in line with bioelectrochemical studies of *P. putida* F1 with exogenous ferricyanide as an electron mediator, which showed a high efficiency of glucose to sugar acid conversion under oxygen-limited BES conditions (Lai et al., 2016).

**TABLE 2:** Summary of glucose and metabolite evaluation of oxygen-limited BES experiments (Data are means of biological duplicates).

**O_2_**	**Strains**	**OD_600_ max**	**Glucose consumption (%)**	**Gluconate (mM)**	**2-Ketogluconate (mM)**	**Acetate (mM)**	**Coulombic efficency (%)**
AA	O1.phz1	1.91	78	0.15	0.89	–	1.34
	O1.phz2	1.72	80	0.29	0.10	0.84	1.47
	14.phz1	1.74	44	0.08	1.12	0.65	1.12
	14.phz2	1.78	87	0.76	0.63	2.90	2.72
	O1.phz1 +	1.90	63	0.59	2.30	1.53	1.45
	O1.phz2 +	1.87	88	0.69	0.07	1.56	1.53
	14.phz1 +	2.25	78	2.90	1.70	2.25	1.77
	14.phz2 +	2.45	83	4.80	1.30	2.45	5.90
PA	O1.phz1	1.21	72	0.07	0.91	–	1.04
	O1.phz2	1.73	68	0.42	0.70	0.99	2.05
	14.phz1	1.02	39	1.91	0.55	1.38	0.77
	14.phz2	1.88	79	0.05	0.47	0.59	5.40
	O1.phz1 +	1.70	65	0.38	0.29	0.63	3.38
	O1.phz2 +	1.71	68	1.50	1.25	–	2.89
	14.phz1 +	1.54	81	0.17	0.29	–	1.45
	14.phz2 +	1.79	46	0.04	1.89	1.46	5.89

The estimation of energy retrieved from the carbon source in BES can be calculated as coulombic efficiency (Sleutels et al., 2011). Overall the coulombic efficiency observed from all strains was low and most of the consumed charge was used for slow respiration with oxygen (Table 2). However, the most active strains *P. putida* 14.phz2 and *P. putida* 14.phz2+ showed a significantly increased coulombic efficiency of up to 6% for both active and PA. This result shows that already small changes in the strain design for phenazine synthesis can not only improve phenazine production but also the efficiency of bioelectrochemical interaction of this strain. For a further improvement of phenazine based electron discharge to the anode, specific knowledge about the involved biochemical and energetic reaction pathways is required. However, this information is not available yet, neither for *P. aeruginosa* nor for *P. putida*. Work is on-going in our group to elucidate the specific phenazine electron transfer pathways and their integration into the energetic pathways of the microorganisms to remove electron discharge bottlenecks.

### Deciphering the Effects of Operon and Strain Origin for Heterologous Production of Phenazine in *P. putida*

**FIGURE 3:**
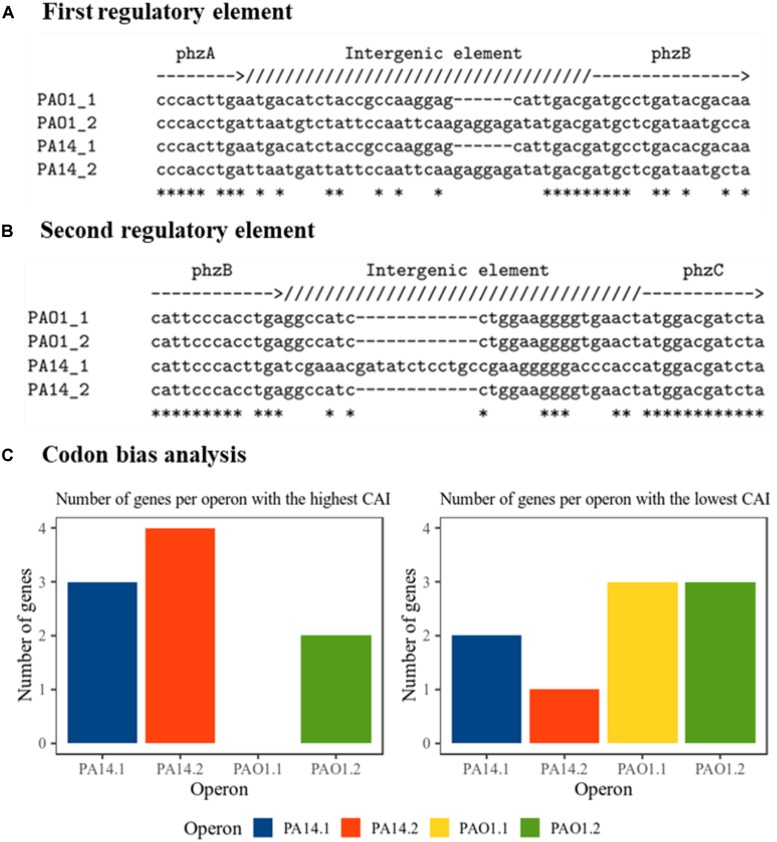
Nucleotide-based analysis of the phenazine clusters. **(A)** First intergenic regulatory element alignment; between *phzA* and *phzB*. Deleted nucleotides are marked as hyphen (-). Identical bases are marked as asterisk (^∗^). **(B)** Second intergenic regulatory element alignment; between *phzB* and *phzC*. Deleted nucleotides are marked as hyphen (-). Identical bases are marked as asterisk (^∗^). **(C)** Prediction of the expression tendency. Bars represent the number of genes in each operon detected with the highest or the lowest codon adaptation indexes, CAI, as detailed in Supplementary Table S2. Genes with equally high or low values are counted double.

To elucidate possible reasons for the difference in phenazine production and resulting BES performance, we took a deeper look at the gene origin for PCA synthesis at DNA and protein level (Supplementary Data S1). In all constructs, we omitted the native promoter region of the operons and cloned the genes in a uniform salicylic acid inducible vector. Thus, any difference in performance and activity should originate from the sequence information of the genes. All plasmids have been re-sequenced to confirm the correctness of the original sequence and exclude mutations. In order to obtain a deeper understanding of the gene characteristic and its impact to dictate the phenazine production, a comparative *in silico* analysis of all genes used in this study has been conducted. Figures 3A,B shows the nucleotide alignment corresponding to two potential regulatory intergenic regions. These regions are long enough to allow for the attachment of DNA-binding proteins and they carry their own ribosome binding sites (consensus sequence: 5′-AGGAGG-3′). The first regulatory element (Figure 3A) is located between the genes *phzA* and *phzB*. The RBS of *phzB* (ribosome binding sites) is slightly interrupted by a deletion in the operons 1 compared with operons 2, where the RBS are complete and more conserved to the consensus sequence. The PhzB protein plays an important role to accelerate the condensation of two molecules of the intermediate product called 6-amino-5-oxocyclohex-2-ene-1-carboxylic acid (AOCHC) in the PCA synthesis (Blankenfeldt and Parsons, 2014). Thus, changes in PhzB numbers will significantly influence overall phenazine synthesis. Thus, a more functional RBS for *phzB* in operons 2 could be reflected in the overall higher phenazine synthesis from these operons compared to operons 1. Furthermore, the second regulatory element (Figure 3B) is located between the genes *phzB* and *phzC*. There is an insertion observed in operon 1 from strain PA14. The RBS in this operon seems to be slightly longer compared to all other operon. Other intergenic regions presenting RBS (Supplementary Data S2) were also detected but no differences observed among operons. Those regions are located between *phzE* and *phzF* as well as between *phzF* and *phz*G.

An important aspect of heterologous gene expression is the fact that every organism is likely to prefer distinctive codons. In the genes of different organisms, a non-random distribution of synonymous codons has been explicated by comparative sequence analysis, the so-called codon bias (Quax et al., 2015). Besides influencing the expression levels of proteins, the codon bias is also affecting protein folding and regulation of protein expression (Pechmann and Frydman, 2013; Gingold et al., 2014). It has been revealed that even among genes in one genome, the codon usage might vary. In order to detect changes between the codon usages among genes in the four operons, codon adaptation indices CAI have been calculated for all genes using an index previously generated for *P. putida* KT2440 (Sharp et al., 2005). Counting the genes in each operon for which the highest (best fit of codon usage) or lowest (worst fit) CAI has been calculated (Figure 3C and Supplementary Table S2) gives an overall idea about the potential expression success of these operons. According to the results, operon 2 of PA14 would tend to have a better expression compared with the other constructs, which corresponds well to the experimental results.

**FIGURE 4:**
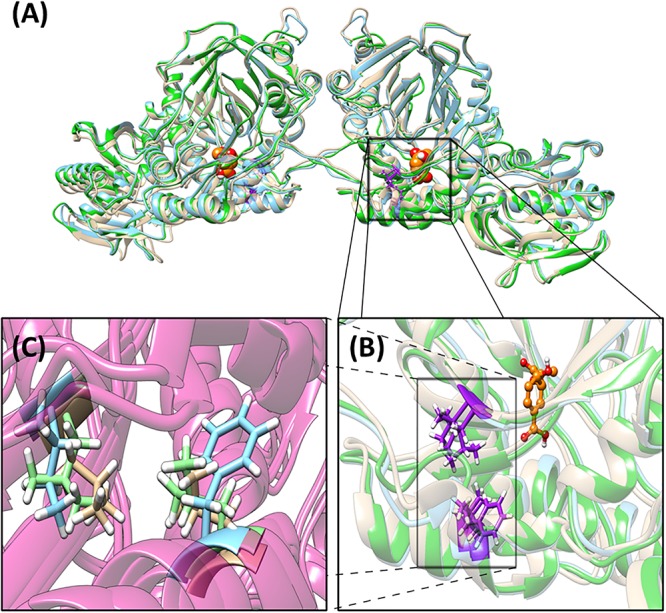
Structural analysis of PhzE. **(A)** PA14-PhzE2 is used as the reference for the alignment (blue). RMSD for PA14-PhzE1 (yellow): 1.056 Å. RMSD for PAO1-PhzE1 (green): 0.836 Å. PAO1-PhzE2 is identical to PAO1-PhzE1. Unique amino acid changes regarding in PA14-PhzE2 [p.(F231L;I266L)] are marked in purple. Substrate (chorismic acid) molecules are marked in yellow. **(B)** Detail of the substrate binding site vicinity. **(C)** Detail of the unique amino acid changes in PA14-PhzE2 and location inside the structure showing the phenylalanine in positon 231 and the isoleucine in position 266 for PA14-PhzE2 (both blue) compared to the leucine substitutions for both positions in the other PhzE proteins (green and yellow).

A phylogenetic tree describing the evolutionary relation between the four operons has been generated (Supplementary Figure S7). The differences between operons within one strain are more prominent compared to the differences between strains. The detailed amino acid comparison between operon 1 and 2, either in PAO1 or PA14, gave the same result (Supplementary Data S1). The overall amino acid alignment analysis showed the biggest difference between operons 1 and 2 in the PhzB protein (91% identical), followed by the PhzA protein (97% identical). However, our protein structure analysis showed that these differences are in amino acids, which are located at the N- and C-terminus on the outer sphere of the protein, and are likely not affecting the protein function. In general, the structural analysis detected mostly small conformation changes in most of the enzymes involved in PCA synthesis (Supplementary Figures S8–S12). These changes are mainly located in the N-terminus, C-terminus and nearby loop regions, which could be the target of post-translational modifications and therefore these regions may not be present in the mature model protein. An exception is the protein PhzE in operon 2 of strain PA14 for which we observed relevant variations, which may be important in the activity of this protein (Figure 4). Structure differences are probably due to conformational changes of this protein resulting from two unique amino acid exchanges located close to each other and close to the enzyme catalytic site where the substrate chorismic acid binds (i.e., position 231 is a phenylalanine in PA14-phzE2 and a leucine for the other three PhzE versions; and position 266 is an isoleucine in PA14-phzE2 and a leucine in the other three PhzE versions). The PhzE protein plays an important role in the phenazine synthesis since it is the first protein of the synthesis pathway. It catalyzes the formation of 2-amino-2-desoxyisochorismic acid (ADIC) from the central metabolic intermediate chorismic acid (Spraggon et al., 2001). Therefore, the specific changes in the active site of this enzyme in the case of PA14-PhzE2 will likely have an impact on the overall phenazine synthesis and might be responsible for the superior performance of the *P. putida* 14.phz2 strain for PCA synthesis observed in this study.

## Conclusion

The activity of heterologous phenazine synthesis in *P. putida* is distinctively influenced by the origin of the genetic information. The gene origin dictates phenazine synthesis rate and yield under fully aerobic shake flask experiments and in oxygen-limited BES conditions. In this study, the *phzA-G* operon 2 from *P. aeruginosa* PA14 (*14.phz2*) was found to result in the best phenazine producer. *P. putida* 14.phz2 stunningly produced 4-times higher PCA concentrations (PCA only producer strain) as well as the respective PCA/PYO amounts (PCA and PYO producer strain) compared to the strains incorporating the other PCA synthesis operons. This elevated phenazine production also resulted in 3-times higher maximum current density and 5-times higher coulombic efficiency than for the other strains. Through deciphering potential sequence based reasons for this superior function, it has been revealed that the *14.phz2* operon contains distinctive regulatory elements for some of the contained genes and a codon usage prediction suggested a best compatibility of operon *14.phz2* with *P. putida*, potentially supporting protein synthesis. Most importantly, for the PhzE2 enzyme of strain PA14, the 3D-protein structure analysis showed two unique amino acid changes located close to the enzyme catalytic site where the substrate chorismic acid binds. This molecular difference might promote higher PCA synthesis with *P. putida* 14.phz2. With these results, *P. putida* 14.phz2 is a promising candidate to be employed as a biocatalyst in an oxygen-limited BES. The anodic electron discharge can now be coupled with the bioelectrochemical production of valuable compounds, such as rhamnolipids. Overall, the presented research findings will be valuable to establish an efficient, oxygen-limited biocatalysis of *P. putida* in BES.

## Data Availability

All datasets generated for this study are included in the manuscript and/or the Supplementary Files.

## Author Contributions

TA planned and conducted the experiments, analyzed the data and wrote the manuscript. SB performed the *in silico* sequence and structure analysis of the phenazine synthesis genes and proteins. LB co-supervised the work, discussed the results and revised the manuscript. MR conceived the work, planned the experiments, discussed the results and revised the manuscript.

## Conflict of Interest Statement

The authors declare that the research was conducted in the absence of any commercial or financial relationships that could be construed as a potential conflict of interest.
