# Supplementary material for: Boosting Heterologous Phenazine Production in Pseudomonas putida KT2440 Through the Exploration of the Natural Sequence Space
Source: Front Microbiol. 2019 Aug 28;10:1990. doi: 10.3389/fmicb.2019.01990 (PMC6722869; doi:10.3389/fmicb.2019.01990)
Supplement: Supplementary file 1 [file Data_Sheet_1.docx]

Supplementary Material

**Boosting heterologous phenazine production in *Pseudomonas putida* KT2440 through the exploration of the natural sequence space**

**Theresia D. Askitosari^1^, Santiago T. Boto^2^, Lars M. Blank^1^, Miriam A. Rosenbaum^2,3^***

^1^Institute of Applied Microbiology IAMB, Aachen Biology and Biotechnology ABBt, RWTH Aachen University, Aachen, Germany

^2^Leibniz Institute for Natural Products Research and Infection Biology – Hans-Knöll-Institute, Jena, Germany

^3^Faculty of Biological Sciences, Friedrich-Schiller-University, Jena, Germany

*corresponding author: [miriam.rosenbaum@leibniz-hki.de](mailto:miriam.rosenbaum@leibniz-hki.de)

# Supplementary Tables, Data, and Figures

This supplementary material contains supplemental tables, figures and data. Specifically it contains details on the primers used in this study (Supplementary Table 1), a supplemental figure showing the BES reactor set up (Supplementary Figure 1), supplemental figures on strain comparison and performance in the multiplexed micro-cultivation (Supplementary Figure 2 & Figure 3), supplemental figures on strain comparison and performance in shake flasks (Supplementary Figure 4 & Figure 5), a supplemental figure of BES cultivation of *P. putida* KT2440 (wildtype) under passive aeration conditions with added synthetic phenazines (Supplementary Figure 6), and supplemental data and figures for our *in silico* gene template analysis (Supplementary Table 2, Supplementary Figure 7 through 13, and Supplementary Data 1 and 2).

## Supplementary Tables

Supplementary Table 1. Primers used for genetic engineering of *P. putida* for phenazine synthesis

| **No** | **Primer** | **Sequence 5’🡪 3’** | **Function** |
| --- | --- | --- | --- |
| 1 | phz2.O1-f | GAAACAGGAGGTACCGCCTCGTCGCCTAGC | amplifying the *phzA2-G2.* PAO1 gene*,* with *Eco*R1 restriction site of overlapping regions for Gibson assembly |
| 2 | phz2.O1-r | CCTCTAGACTCGAGGGCGGTTGGATGGGTTC |  |
| 3 | phz1.14-f | CCGACGTCGCATGCTCCTTGCGGTTATCCGCCATGAAAC | amplifying the *phzA1-G1.* PA14 gene*,* with *Eco*R1 & *Xba*1 restriction site of overlapping regions for Gibson assembly |
| 4 | phz1.14-r | AGGAAACAGGAGGTACCGTCCTACGTATGAACAATGCGC |  |
| 5 | phz2.14-f | GCCCGACGTCGCATGCTCCTGGGCTCCAAGGCCGCGTAG | amplifying the *phzA2-G2.* PA14 gene*,* with *Eco*R1 & *Xba*1 restriction site of overlapping regions for Gibson assembly |
| 6 | phz2.14-r | ACAGGAAACAGGAGGTACCGGGTTACAACTGGGTTTCAGGCGAAAC |  |
| 7 | CP-f | GTCAACGCGAACATTTCC | amplifying the part of *phzG* gene to ORI region of pBNT plasmid (colony PCR verification) |
| 8 | CP-r | CAACTGTTGGGAAGGGCGATCGGTG |  |
| 9 | Seq_1 | GCACCGGACTCCATATC | sequencing part of *phzG* gene to ORI region of pBNT plasmid |
| 10 | Seq_2 | CACCATGCGAGAGTACC | sequencing part of ORI to Kanamycin resistance cassette of pBNT plasmid |
| 11 | Seq_3 | GCTGTGGCGGTTTATGG | sequencing part of *nagR*/*pNagAa* promoter region of pBNT plasmid to part of *phzA* gene |
| 12 | Seq_4 | GATCCTCAAGGGCTATG | sequencing part of *phzA* gene to part of *phzB* gene |
| 13 | Seq_5 | GCGGCATTCCCGAAATC | sequencing part of *phzB* gene to part of *phzC* gene |
| 14 | Seq_6 | GTTCGCCTTGCTCTACC | sequencing part of *phzC* gene |
| 15 | Seq_7 | CGACAACCGCAAGGAAG | sequencing part of *phzC* gene to part of *phzD* gene |
| 16 | Seq_8 | CGATGATCGCCAAGCAG | sequencing part of *phzD* gene to part of *phzE* gene |
| 17 | Seq_9 | CGCATCCGCATCTTCAC | sequencing part of *phzE* gene |
| 18 | Seq_10 | CCATGATGGGCGTCAAC | sequencing part of *phzE* gene to part of *phzF* gene |
| 19 | Seq_11 | CACTGGCAGAGCATTAC | sequencing part of *phzF* gene to part of *phzG* gene |

**Supplementary Table 2. Codon Adaptation Index for all genes belonging to the four clusters of interest**. This index is a measurement to predict the level of expression of a gene taking into account the codon usage, being 1 the best expressed and 0 the worst expressed.


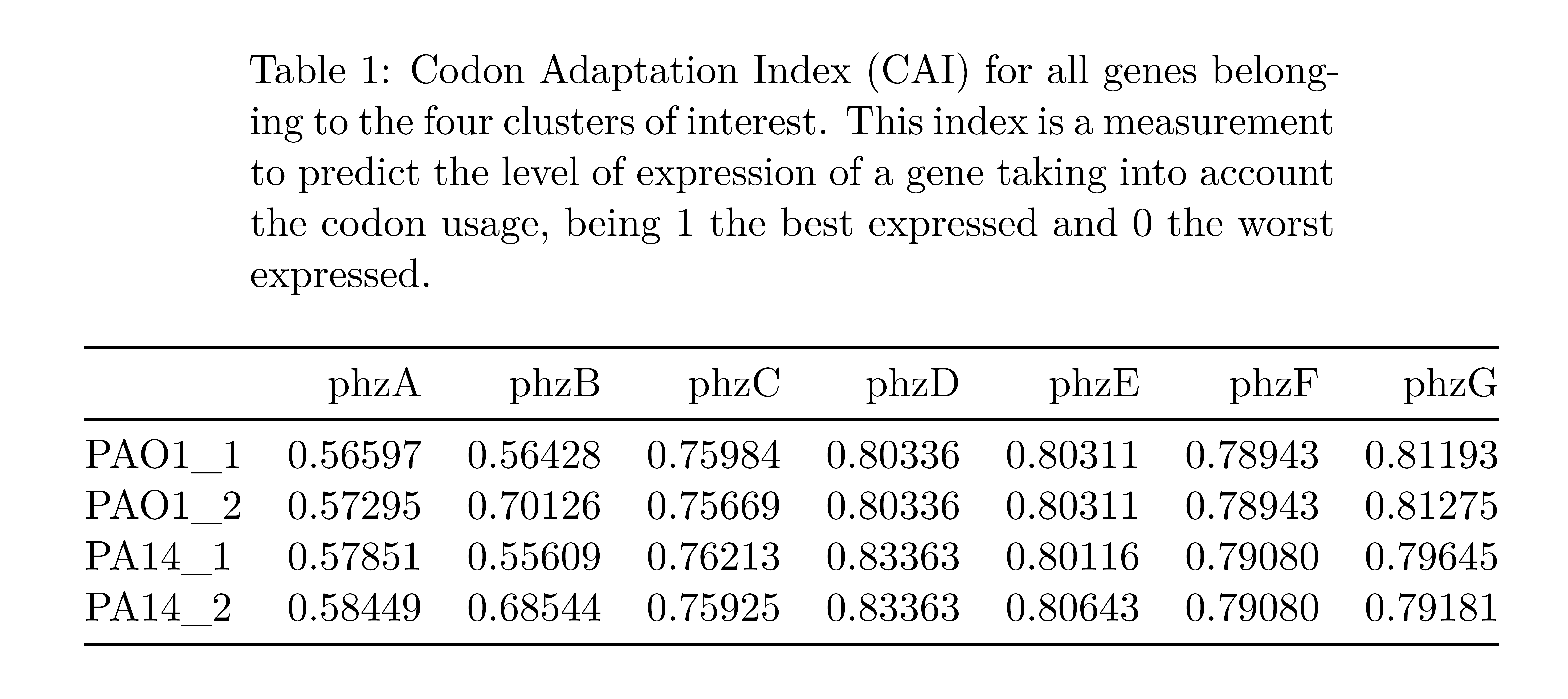


## Supplementary Figures


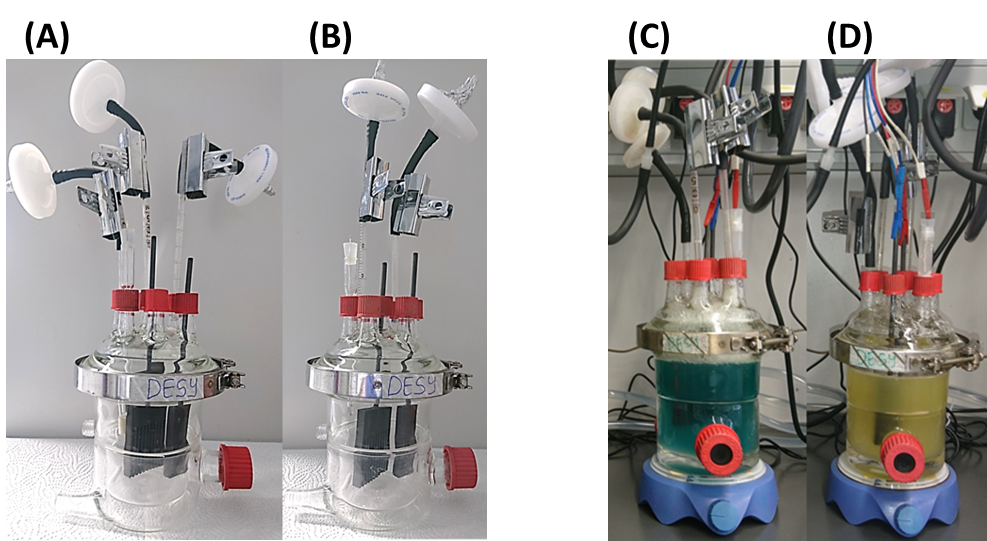


**Supplementary Figure 1. Images of bioelectrochemical reactors used in this study (A)** Active aeration set-up **(B)** Passive aeration set-up **(C)** *P. putida* 14.phz2+ produced PCA and PYO indicated by blue pigment in the grown culture **(D)** *P. putida* 14.phz2 produced PCA indicated by yellow pigment in the grown culture.


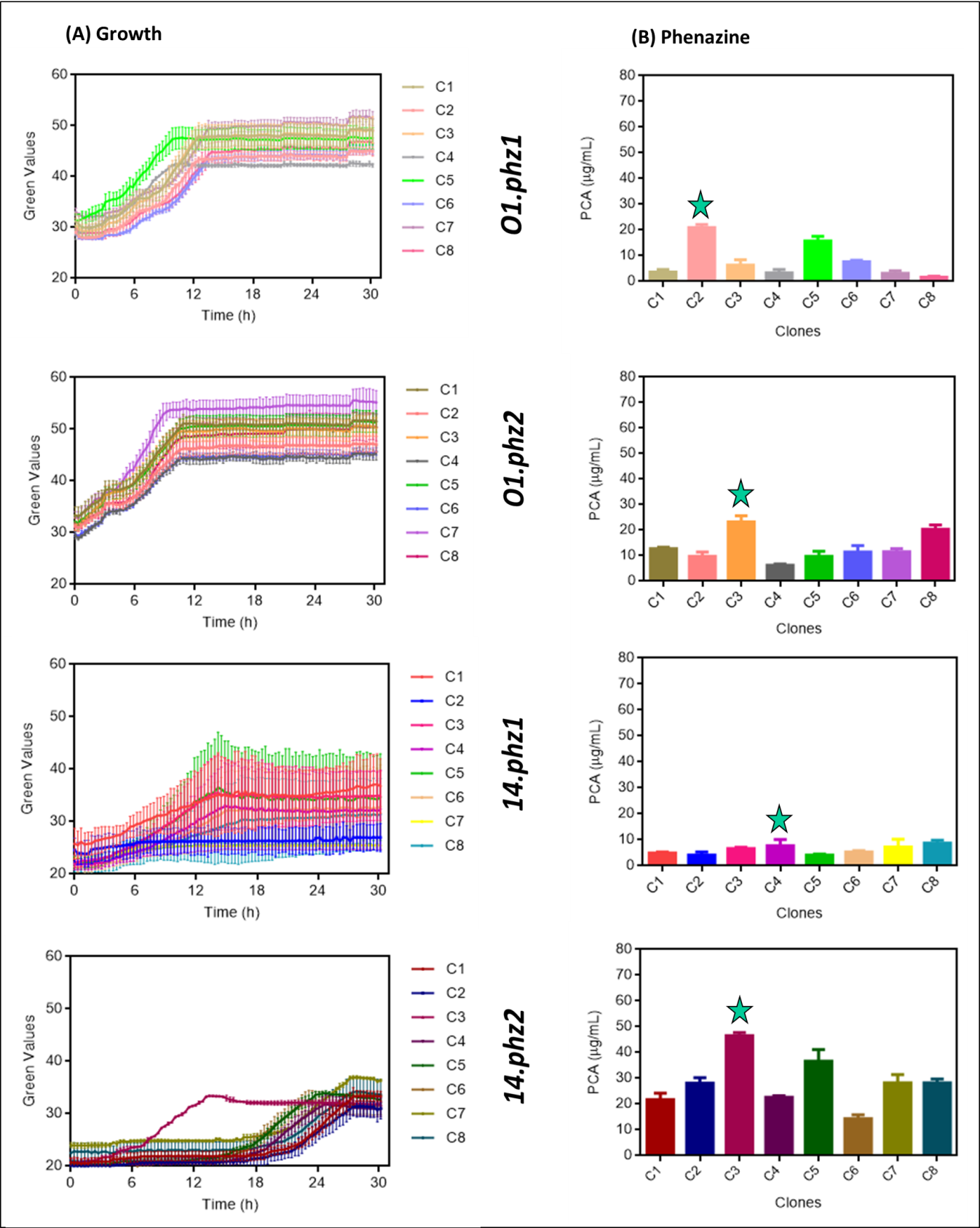


Supplementary Figure 2. Growth curve (A) and PCA production (B) of eight individual clones of *P. putida* O1.phz1, O1.phz2, 14.phz1, and 14.phz2 in the micro-cultivation experiment (triplicates for each). For this cultivation platform the “green value” is the output signal for biomass density. The star symbol indicates the selected clone to be characterized in the subsequent flask & BES experiment.


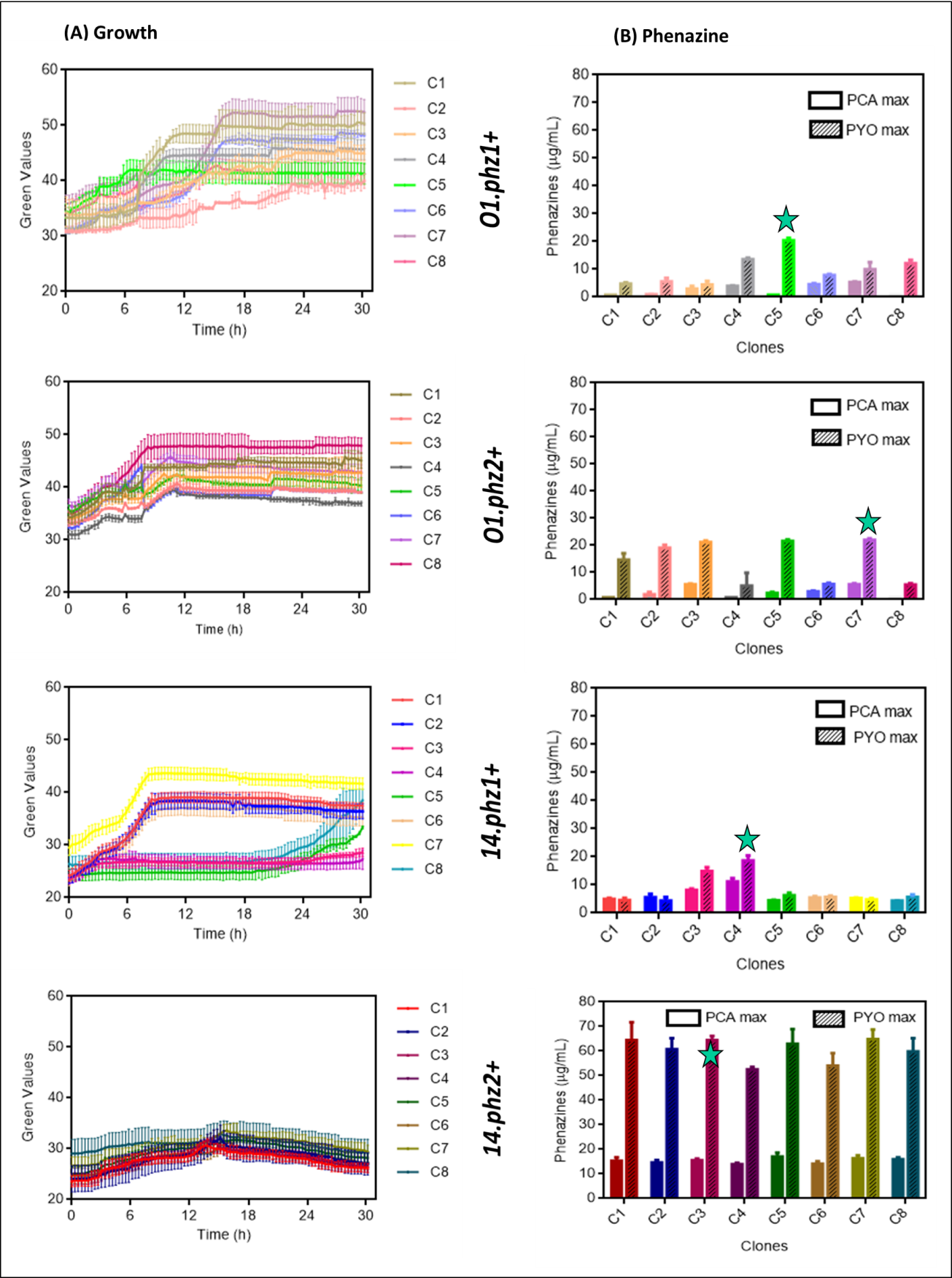


Supplementary Figure 3. Growth curve (A) and PCA & PYO production (B) of eight clones of *P. putida* O1.phz1+, O1.phz2+, 14.phz1+, and 14.phz2+ in the micro-cultivation experiment (triplicates for each). For this cultivation platform the “green value” is the output signal for biomass density. The star symbol indicates the selected clone to be characterized in the subsequent flask & BES experiment.


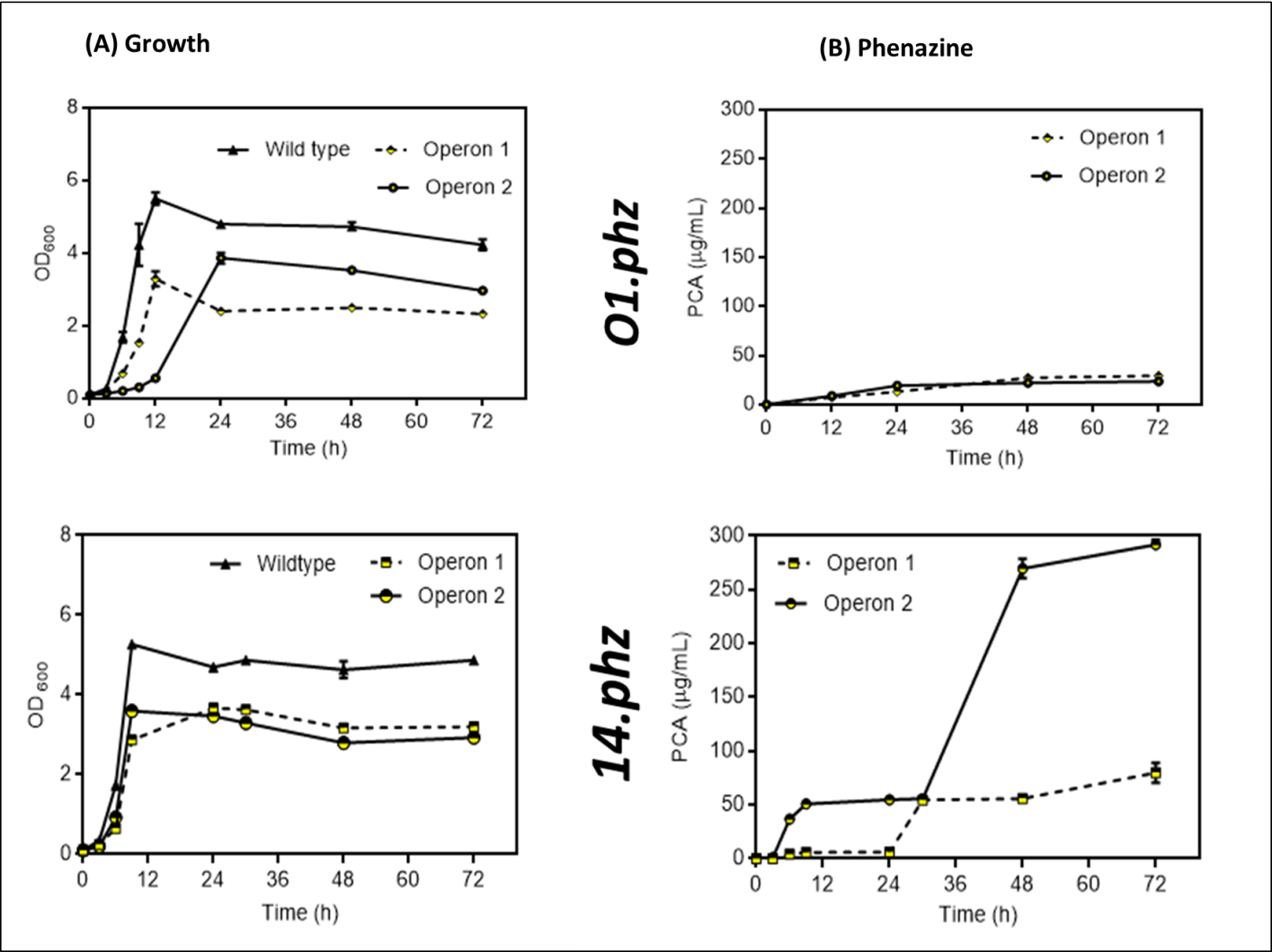


**Supplementary Figure 4. Growth curve (A) and PCA production (B)** of the selected *P. putida* O1.phz1, O1.phz2 *(both top graphs),* 14.phz1, and 14.phz2 (both bottom graphs) clones in the shake flask experiment (triplicates).


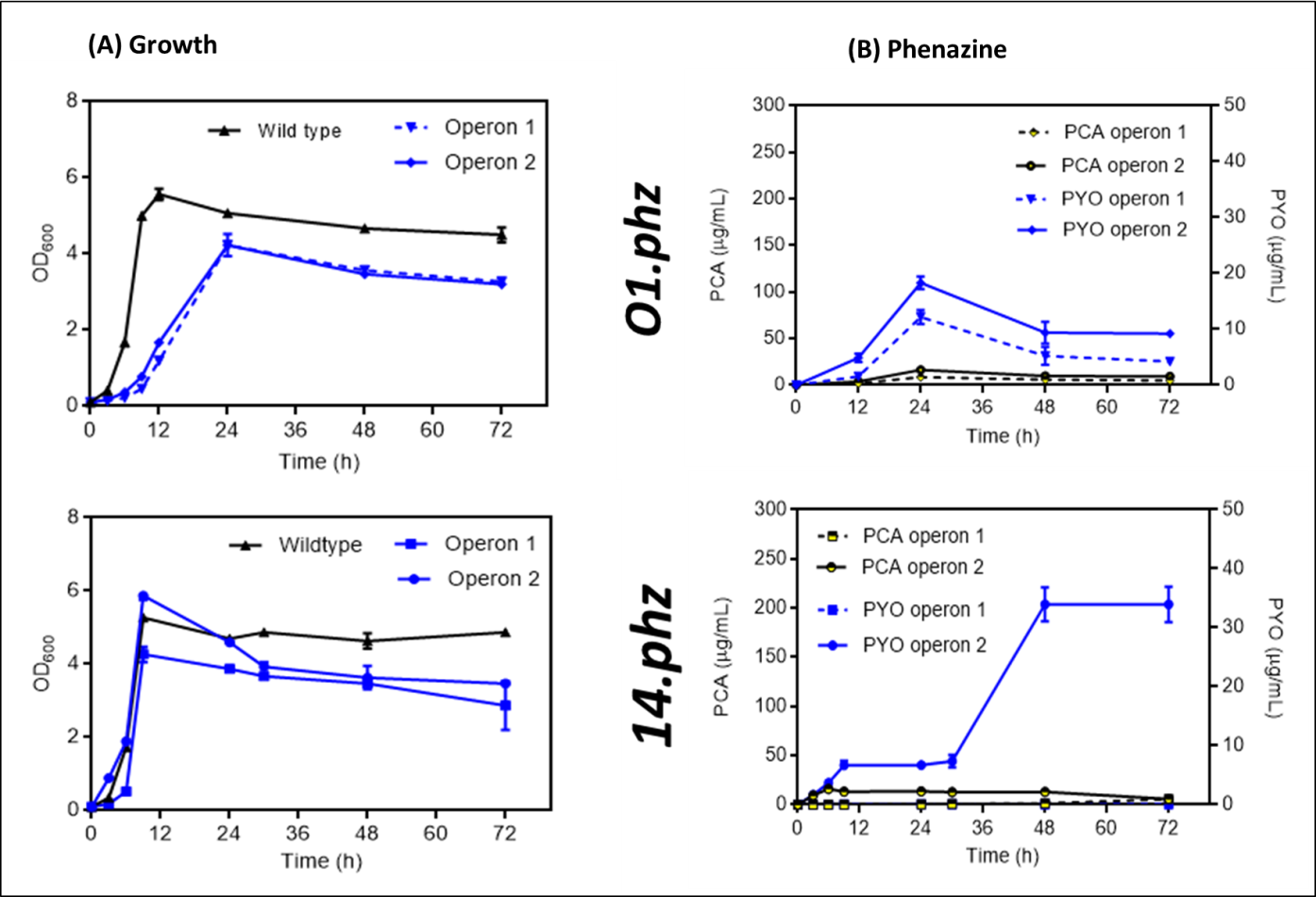


**Supplementary Figure 5. Growth curve (A) and PCA & PYO production (B)** of the selected *P. putida* O1.phz1+, O1.phz2+ (both top graphs), 14.phz1+, and 14.phz2+ (both bottom graphs) clones in the shake flask experiments (triplicates).


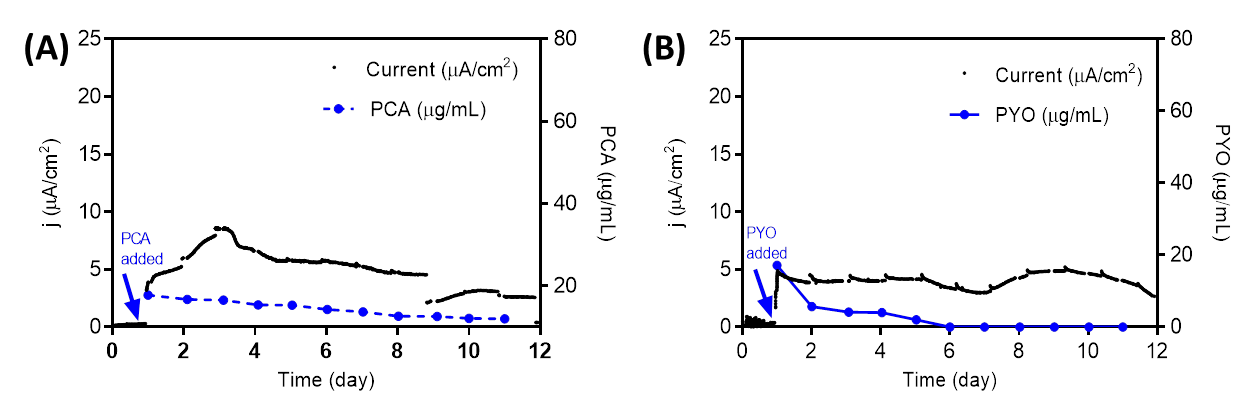


**Supplementary Figure 6. BES cultivation of *P. putida* KT2440 (wildtype) under passive aeration conditions**. Synthetic PCA **(A)** or PYO **(B)** was added (~17µg/mL) after 24 hours inoculation (day 1).

**
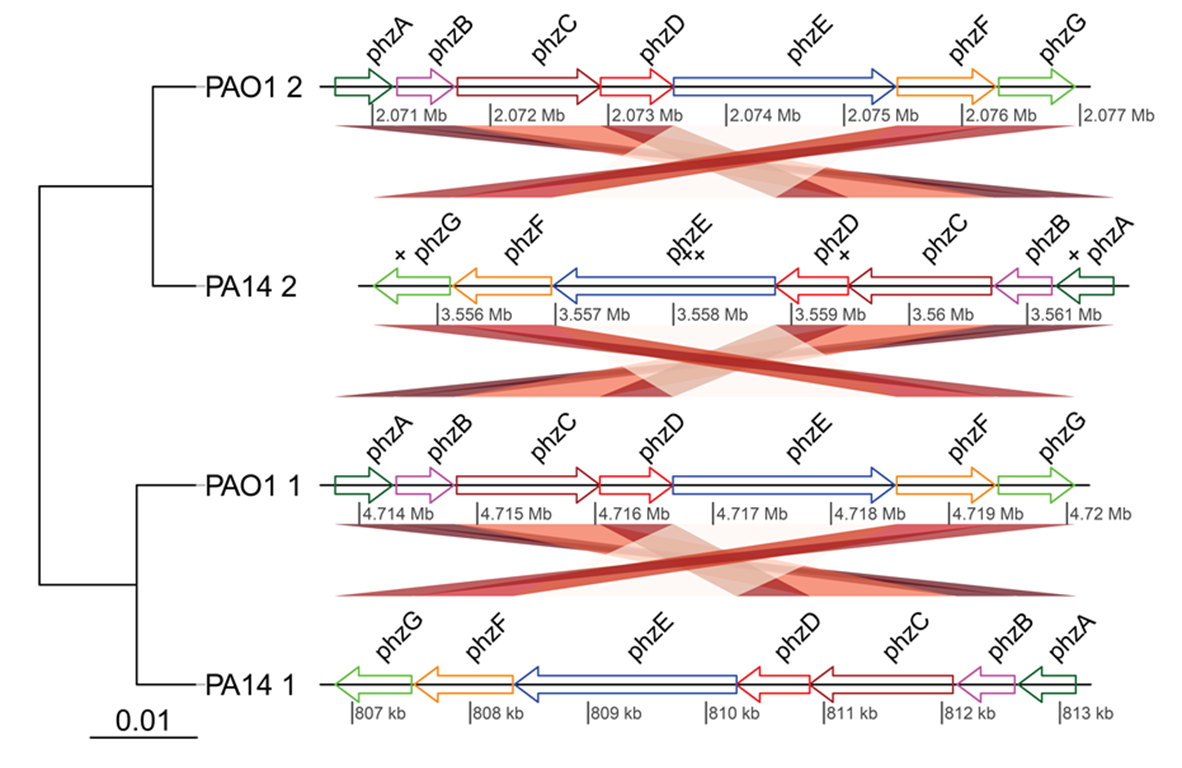
**

**Supplementary Figure 7. Phylogenetic tree and genomic plot of the phenazine production operons.** Links between homology genes are represented with red shading where the intensity of the colour is decreasing with the length of the gene. Unique amino acid changes are marked with an x in the best phenazine-producing operon (PA14.2).


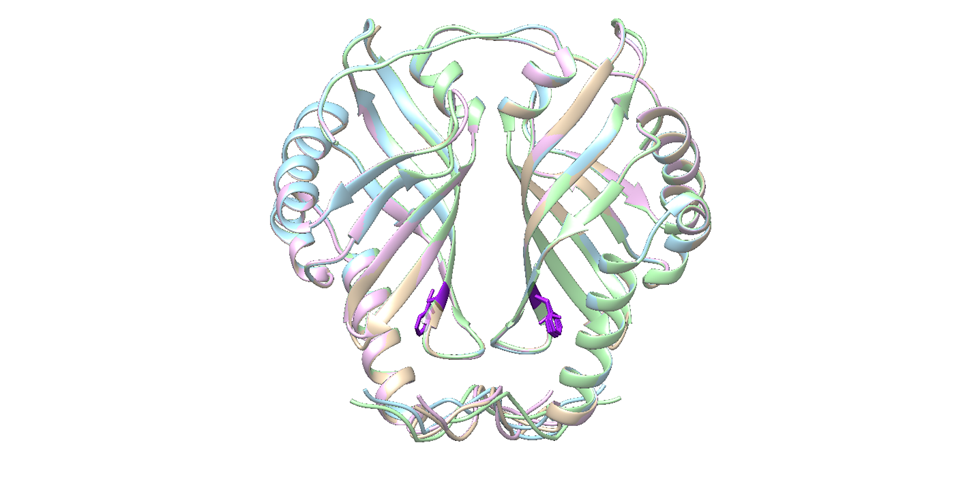


**Supplementary Figure 8. Structural alignment PhzA.** PA14-PhzA2 is used as the reference for the alignment (blue). RMSD for PA14-PhzA1 (pink): 0.177 Å. RMSD for PAO1-PhzA1 (light purple): 0.141 Å. RMSD for PAO1-PhzA2 (green): 0.249 Å. Unique amino acid changes regarding PA14-PhzA2 (p.L97F) are marked in purple.


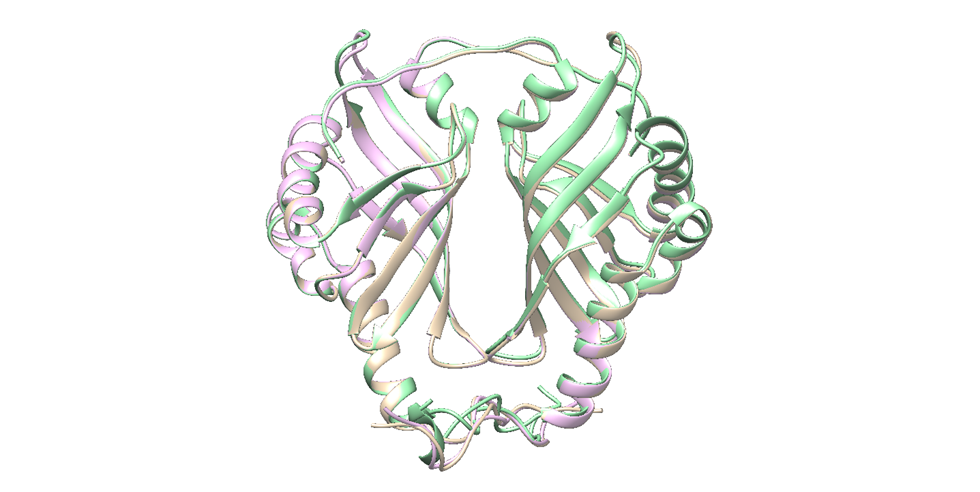


**Supplementary Figure 9. Structural alignment PhzB.** PA14-PhzB2 is used as the reference for the alignment (green). RMSD for PA14-PhzB1 (pink): 0.277 Å. RMSD for PAO1-PhzB1 (light purple): 0.348 Å. PAO1-PhzB2 is identical to PA14-PhzB2.


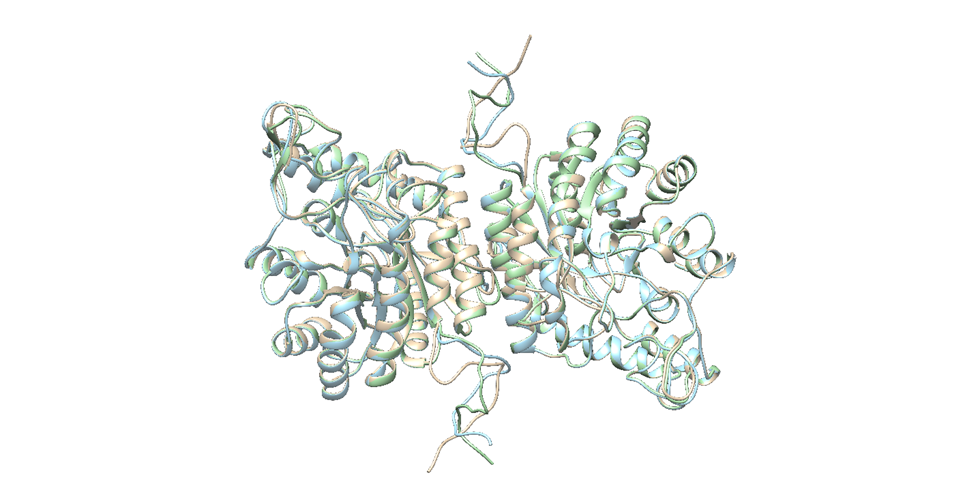


**Supplementary Figure 10. Structural alignment PhzC.** PA14-PhzC2 is used as the reference for the alignment (blue). RMSD for PA14-PhzC1 (pink): 0.185 Å. RMSD for PAO1-PhzC1 (green): 0.209 Å. PAO1-PhzC2 is identical to PAO1-PhzC1.


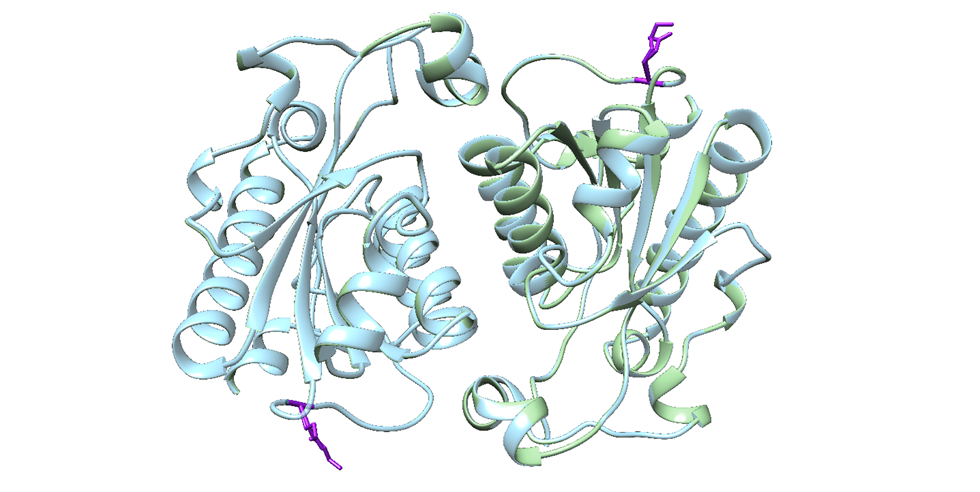


**Supplementary Figure 11. Structural alignment PhzD.** PA14-PhzD2 is used as the reference for the alignment (blue). RMSD for PA14-PhzD1 (green): 0.059 Å. PAO1-PhzD1 and PAO1-PhzD2 are identical to PA14-PhzD1. Unique amino acid changes regarding PA14-PhzD2 (p.K28E) are marked in purple.


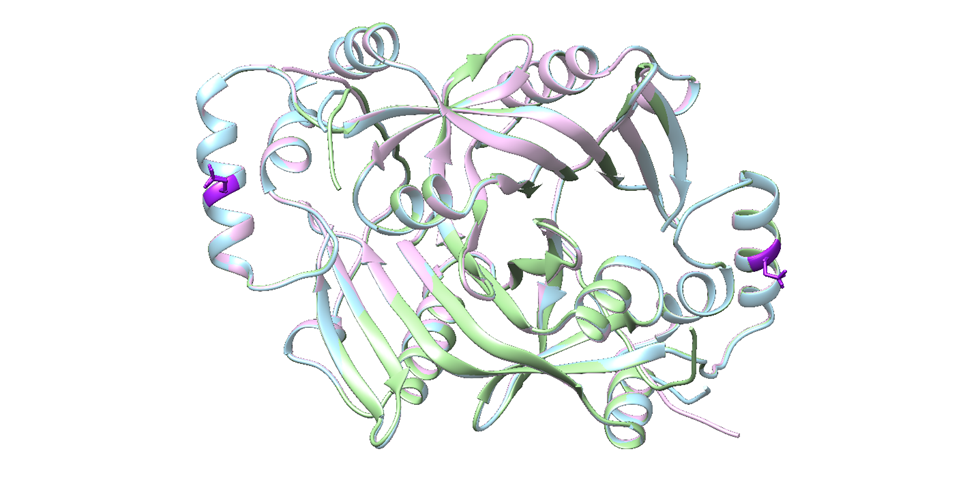


**Supplementary Figure 12. Structural alignment PhzG.** PA14-PhzG2 is used as the reference for the alignment (blue). RMSD for PA14-PhzG1 (green): 0.231 Å. RMSD for PAO1-PhzG1 (light purple): 0.159 Å. PAO1-PhzG2 is identical to PA14-PhzG1. Unique amino acid changes regarding PA14-PhzG2 (p.A158E) are marked in purple.


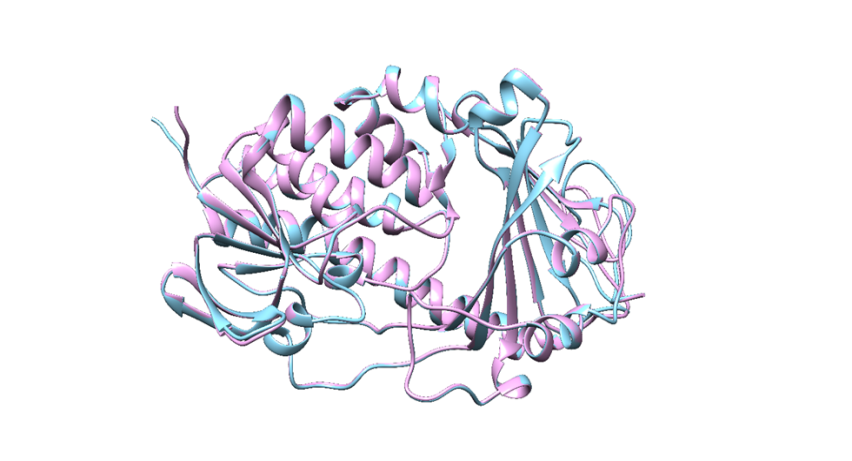


**Supplementary Figure 13. Structural alignment PhzS.** PA14-PhzS is used as the reference for the alignment (blue). RMSD for PAO1‑PhzS (purple): 0.178 Å.

## Supplementary Data

## *In silico* phenazine gene origin analysis

**Supplementary Data 1. Protein alignments**

According to Clustal documentation (http://www.clustal.org/omega/), the meaning of the symbols in the output is the following (simplified):

- Asterisk: Conserved position
- Colon: Strongly similar properties (only for amino acids)
- Period: Weakly similar properties (only for amino acids)

The following UNIX command has been used with different inputs to perform all the alignments:

clustalo -i input_sequences.fasta -o output_alignment --outfmt clu

--resno --force --guidetree-out output_tree

Alignment for PhzA:

CLUSTAL O(1.2.4) multiple sequence alignment

PAO1_1 MNGQRYRETPLDIERLRRLNRATVERYMAMKGAERLQRHSLFVEDGCAGNWTTESGEPLV 60

PAO1_2 MREYQRLKGFTDNLELRRRNRATVEHYMRMKGAERLQRHSLFVEDGCAGNWTTESGEPLV 60

PA14_1 MNGQRYRETPLDIERLRRLNRATVERYMAMKGAERLQRHSLFVEDGCAGNWTTESGEPLV 60

PA14_2 MREYQRLKGFTDNLELRRRNRATVEHYMRMKGAERLQRHSLFVEDGCAGNWTTESGEPLV 60

*. : : * .*** ******:** *******************************

PAO1_1 FRGHESLRRLAEWLERCFPDWEWHNVRIFETEDPNHFWVECDGRGKALVPGYPQGYCENH 120

PAO1_2 FRGHESLRRLAEWLERCFPDWEWHNVRIFETEDPNHFWVECDGRGKALVPGYPQGYCENH 120

PA14_1 FRGHESLRRLAEWLERCFPDWEWRNVRIFETEDPNHFWVECDGRGKALVPGYPQGYCENH 120

PA14_2 FRGHESLRRLAEWLERCFPDWEWHNVRIFETEDPNHLWVECDGRGKALVPGYPQGYCENH 120

***********************:************:***********************

PAO1_1 YIHSFELENGRIKRNREFMNPIQKLRALGIAVPQIKRDGIPT 162

PAO1_2 YIHSFELENGRIKRNREFMNPMQKLRALGIAVPQIKRDGIPT 162

PA14_1 YIHSFELENGRIKRNREFMNPMQKLRALGIAVPQIKRDGIPT 162

PA14_2 YIHSFELENGRIKRNREFMNPMQKLRALGIAVPQIKRDGIPT 162

*********************:********************

Alignment for PhzB:

CLUSTAL O(1.2.4) multiple sequence alignment

PAO1_1 MPDTTNPIGFTDANELREKNRATVEKYMNTKGQDRLRRHELFVEDGCGGLWTTDTGSPIV 60

PAO1_2 MLDNAIPQGFEDAVELRRKNRETVVKYMNTKGQDRLRRHELFVEDGCGGLWTTDTGSPIV 60

PA14_1 MPDTTNPIGFTDANELREKNRATVEKYMNTKGQDRLRRHELFVEDGCGGLWTTDTGSPIV 60

PA14_2 MLDNAIPQGFEDAVELRRKNRETVVKYMNTKGQDRLRRHELFVEDGCGGLWTTDTGSPIV 60

* *.: * ** ** ***.*** ** ***********************************

PAO1_1 IRGKDKLAEHAVWSLKCFPDWEWYNINIFGTDDPNHFWVECDGHGKILFPGYPEGYYENH 120

PAO1_2 IRGKDKLAEHAVWSLKCFPDWEWYNIKVFETDDPNHFWVECDGHGKILFPGYPEGYYENH 120

PA14_1 IRGKDKLAEHAVWSLKCFPDWEWYNINIFGTDDPNHFWVECDGHGKILFPGYPEGYYENH 120

PA14_2 IRGKDKLAEHAVWSLKCFPDWEWYNIKVFETDDPNHFWVECDGHGKILFPGYPEGYYENH 120

**************************::* ******************************

PAO1_1 FLHSFELEDGKIKRNREFMNVFQQLRALSIPVPQIKREGIPT 162

PAO1_2 FLHSFELDDGKIKRNREFMNVFQQLRALSIPVPQIKREGIPT 162

PA14_1 FLHSFELEDGKIKRNREFMNVFQQLRALSIPVPEIKREGIPT 162

PA14_2 FLHSFELDDGKIKRNREFMNVFQQLRALSIPVPQIKREGIPT 162

*******:*************************:********

Alignment for PhzC:

CLUSTAL O(1.2.4) multiple sequence alignment

PAO1_1 MDDLLQRVRRCEALQQPEWGDPSRLRDVQAYLRGSPALIRAGDILALRATLARVARGEAL 60

PAO1_2 MDDLLQRVRRCEALQQPEWGDPSRLRDVQAYLRGSPALIRAGDILALRATLARVARGEAL 60

PA14_1 MDDLLQRVRRCEALQQPEWGDPSRLRDVQAYLRGSPALIRAGDILALRATLARVARGEAL 60

PA14_2 MDDLLQRVRRCEALQQPEWGDPSRLRDVQAYLRGSPALIRAGDILALRATLARVARGEAL 60

************************************************************PAO1_1 VVQCGDCAEDMDDHHAENVARKAAVLELLAGALRLAGRRPVIRVGRIAGQYAKPRSKPHE 120

PAO1_2 VVQCGDCAEDMDDHHAENVARKAAVLELLAGALRLAGRRPVIRVGRIAGQYAKPRSKPHE 120

PA14_1 VVQCGDCAEDMDDHHAENVARKAAVLELLAGALRLAGRRPVIRVGRIAGQYAKPRSKPHE 120

PA14_2 VVQCGDCAEDMDDHHAENVARKAAVLELLAGALRLAGRRPVIRVGRIAGQYAKPRSKPHE 120

************************************************************

PAO1_1 QVGEQTLPVYRGDMVNGREAHAEQRRADPQRILKGYAAARNIMRHLGWDAASGQEANASP 180

PAO1_2 QVGEQTLPVYRGDMVNGREAHAEQRRADPQRILKGYAAARNIMRHLGWDAASGQEANASP 180

PA14_1 QVGEQTLPVYRGDMVNGREAHAEQRRADPQRILKGYAAARNIMRHLGWDAASGQEANASP 180

PA14_2 QVGEQTLPVYRGDMVNGREAHAEQRRADPQRILKGYAAARNIMRHLGWDAASGQEANASP 180

************************************************************

PAO1_1 VWTSHEMLLLDYELSMLREDEQRRVYLGSTHWPWIGERTRQVDGAHVALLAEVLNPVACK 240

PAO1_2 VWTSHEMLLLDYELSMLREDEQRRVYLGSTHWPWIGERTRQVDGAHVALLAEVLNPVACK 240

PA14_1 VWTSHEMLLLDYELSMLREDEQRRVYLGSTHWPWIGERTRQVDGAHVALLAEVLNPVACK 240

PA14_2 VWTSHEMLLLDYELSMLREDEQRRVYLGSTHWPWIGERTRQVDGAHVALLAEVLNPVACK 240

************************************************************

PAO1_1 VGPEIGRDQLLALCERLDPRREPGRLTLIARMGAQKVGERLPPLVEAVRAAGHPVIWLSD 300

PAO1_2 VGPEIGRDQLLALCERLDPRREPGRLTLIARMGAQKVGERLPPLVEAVRAAGHPVIWLSD 300

PA14_1 VGPEIGRDQLLALCERLDPRREPGRLTLIARMGAQKVGERLPPLVEAVRAAGHPVIWLSD 300

PA14_2 VGPEIGRDQLLALCERLDPRREPGRLTLIARMGAQKVGERLPPLVEAVRAAGHPVIWLSD 300

************************************************************

PAO1_1 PMHGNTIVAPCGNKTRLVRSIAEEVAAFRLAVSGSGGVAAGLHLETTPDDVTECVADSSG 360

PAO1_2 PMHGNTIVAPCGNKTRLVRSIAEEVAAFRLAVSGSGGVAAGLHLETTPDDVTECVADSSG 360

PA14_1 PMHGNTIVAPCGNKTRLVRSIAEEVAAFRLAVSGSGGVAAGLHLETTPDDVTECVADSSG 360

PA14_2 PMHGNTIVAPCGNKTRLVRSIAEEVAAFRLAVSGSGGVAAGLHLETTPDDVTECVADSSG 360

************************************************************

PAO1_1 LHQVSRHYTSLCDPRLNPWQALSAVMAWSGAEAIPSATFPLETVA 405

PAO1_2 LHQVSRHYTSLCDPRLNPWQALSAVMAWSGAEAIPSATFPLETVA 405

PA14_1 LHQVGRHYTSLCDPRLNPWQALSAVMAWAGAEATPSATFPLETVA 405

PA14_2 LHQVSRHYTSLCDPRLNPWQALSAVMAWAGAEAIPSATFPLETVA 405

****.***********************:**** ***********

Alignment for PhzD:

CLUSTAL O(1.2.4) multiple sequence alignment

PAO1_1 MSGIPEITAYPLPTAQQLPANLARWSLEPRRAVLLVHDMQRYFLRPLPESLRAGLVANAA 60

PAO1_2 MSGIPEITAYPLPTAQQLPANLARWSLEPRRAVLLVHDMQRYFLRPLPESLRAGLVANAA 60

PA14_1 MSGIPEITAYPLPTAQQLPANLARWSLEPRRAVLLVHDMQRYFLRPLPESLRAGLVANAA 60

PA14_2 MSGIPEITAYPLPTAQQLPANLARWSLKPRRAVLLVHDMQRYFLRPLPESLRAGLVANAA 60

***************************:********************************

PAO1_1 RLRRWCVEQGVQIAYTAQPGSMTEEQRGLLKDFWGPGRASPADREVVEELAPGPDDWLL 120

PAO1_2 RLRRWCVEQGVQIAYTAQPGSMTEEQRGLLKDFWGPGRASPADREVVEELAPGPDDWLL 120

PA14_1 RLRRWCVEQGVQIAYTAQPGSMTEEQRGLLKDFWGPGRASPADREVVEELAPGPDDWLL 120

PA14_2 RLRRWCVEQGVQIAYTAQPGSMTEEQRGLLKDFWGPGRASPADREVVEELAPGPDDWLL 120

************************************************************

PAO1_1 TKWRYSAFFHSDLLQRMRAAGRDQLVLCGVYAHVGVLISTVDAYSNDIQPFLVADAIADF 180

PAO1_2 TKWRYSAFFHSDLLQRMRAAGRDQLVLCGVYAHVGVLISTVDAYSNDIQPFLVADAIADF 180

PA14_1 TKWRYSAFFHSDLLQRMRAAGRDQLVLCGVYAHVGVLISTVDAYSNDIQPFLVADAIADF 180

PA14_2 TKWRYSAFFHSDLLQRMRAAGRDQLVLCGVYAHVGVLISTVDAYSNDIQPFLVADAIADF 180

************************************************************

PAO1_1 SEAHHRMALEYAASRCAMVVTTDEVLE 207

PAO1_2 SEAHHRMALEYAASRCAMVVTTDEVLE 207

PA14_1 SEAHHRMALEYAASRCAMVVTTDEVLE 207

PA14_2 SEAHHRMALEYAASRCAMVVTTDEVLE 207

***************************

Alignment for PhzE:

CLUSTAL O(1.2.4) multiple sequence alignment

PAO1_1 MNALPTSLLQRLLERPAPFALLYRPESNGPGLLDVIRGEALELHGLADLPLDEPGPGLPR 60

PAO1_2 MNALPTSLLQRLLERPAPFALLYRPESNGPGLLDVIRGEALELHGLADLPLDEPGPGLPR 60

PA14_1 MNALPTSLLQRLLERPAPFALLYRPESNGPGLLDVIRGETLELHGLADLPLDEPGPGLPR 60

PA14_2 MNALPTSLLQRLLERPAPFALLYRPESNGPGLLDVIRGETLELHGLADLPLDEPGPGLPR 60

***************************************:********************

PAO1_1 HDLLALIPYRQIAERGFEALDDGTPLLALKVLEQELLPLEQALALLPNQALELSEEGFDL 120

PAO1_2 HDLLALIPYRQIAERGFEALDDGTPLLALKVLEQELLPLEQALALLPNQALELSEEGFDL 120

PA14_1 HDLLALIPYRQIAERGFEALDDGTPLLALKVLEQELLPLEQALALLPNQALELSEEAFDL 120

PA14_2 HDLLALIPYRQIAERGFEALDDGTPLLALKVLEQELLPLEQALALLPNQALELSEEAFDL 120

********************************************************.***

PAO1_1 DDEAYAEVVGRVIADEIGRGEGANFVIKRRFQARIDGYATASALSFFRQLLLREKGAYWT 180

PAO1_2 DDEAYAEVVGRVIADEIGRGEGANFVIKRRFQARIDGYATASALSFFRQLLLREKGAYWT 180

PA14_1 DDEAYAEVVGRVIADEIGRGEGANFVIKRRFQARIDGYATASALSFFRQLLLREKGAYWT 180

PA14_2 DDEAYAEVVGRVIADEIGRGEGANFVIKRRFQARIDGYATASALSFFRQLLLREKGAYWT 180

************************************************************

PAO1_1 FIVHTGERTLVGASPERHISVRDGLAVMNPISGTYRYPPAGPNLAEVMEFLDNRKEADEL 240

PAO1_2 FIVHTGERTLVGASPERHISVRDGLAVMNPISGTYRYPPAGPNLAEVMEFLDNRKEADEL 240

PA14_1 FIVHTGERTLVGASPERHISVRDGLAVMNPISGTYRYPPAGPNLAEVMEFLDNRKEADEL 240

PA14_2 FIVHTGERTLVGASPERHISVRDGLAVMNPISGTYRYPPAGPNLAEVMEFFDNRKEADEL 240

**************************************************:*********

PAO1_1 YMVVDEELKMARICEDGGRVLGPYLKEMAHLAHTEYFIEGQTSRDVREVLRETLFAPTV 300

PAO1_2 YMVVDEELKMARICEDGGRVLGPYLKEMAHLAHTEYFIEGQTSRDVREVLRETLFAPTV 300

PA14_1 YMVVDEELKMARICEDGGRVLGPYLKEMAHLAHTEYFIEGQTSRDVREVLRETLFAPTV 300

PA14_2 YMVVDEELKMARICEDGGRVLGPYIKEMAHLAHTEYFIEGQTSRDVREVLRETLFAPTV 300

*************************:**********************************

PAO1_1 TGSPLESACRVIRRYEPQGRGYYSGVAALIGGDGQGGRTLDSAILIRTAEIEGDGRLRIG 360

PAO1_2 TGSPLESACRVIRRYEPQGRGYYSGVAALIGGDGQGGRTLDSAILIRTAEIEGDGRLRIG 360

PA14_1 TGSPLESACRVIRRYEPQGRGYYSGVAALIGGDGQGGRTLDSAILIRTAEIESDGRLRIG 360

PA14_2 TGSPLESACRVIRRYEPQGRGYYSGVAALIGGDGQGGRTLDSAILIRTAEIESDGRLRIG 360

****************************************************.*******

PAO1_1 VGSTIVRHSDPLGEAAESRAKASGLIAALKSQAPQRLGSHPHVVAALASRNAPIADFWLR 420

PAO1_2 VGSTIVRHSDPLGEAAESRAKASGLIAALKSQAPQRLGSHPHVVAALASRNAPIADFWLR 420

PA14_1 VGSTIVRHSDPLGEAAESRAKASGLIAALKSQAPQRLGSHPHVVAALASRNAPIADFWLR 420

PA14_2 VGSTIVRHSDPLGEAAESRAKASGLIAALKSQAPQRLGSHPHVVAALASRNAPIADFWLR 420

************************************************************

PAO1_1 GASERQQLQADLSGREVLIVDAEDTFTSMIAKQLKSLGLTVTVRGFQEPYSFDGYDLVIM 480

PAO1_2 GASERQQLQADLSGREVLIVDAEDTFTSMIAKQLKSLGLTVTVRGFQEPYSFDGYDLVIM 480

PA14_1 GASERQQLQADLSGREVLIVDAEDTFTSMIAKQLKSLGLTVTVRGFQEPYSFDGYDLVIM 480

PA14_2 GASERQQLQADLSGREVLIVDAEDTFTSMIAKQLKSLGLTVTVRGFQEPYSFDGYDLVIM 480

************************************************************

PAO1_1 GPGPGNPTEIGQPKIGHLHLAIRSLLSERRPFLAVCLSHQVLSLCLGLDLQRRQEPNQGV 540

PAO1_2 GPGPGNPTEIGQPKIGHLHLAIRSLLSERRPFLAVCLSHQVLSLCLGLDLQRRQEPNQGV 540

PA14_1 GPGPGNPTEIGQPKIGHLHLAIRSLLSERRPFLAVCLSHQVLSLCLGLDLQRRQEPNQGV 540

PA14_2 GPGPGNPTEIGQPKIGHLHLAIRSLLSERRPFLAVCLSHQVLSLCLGLDLQRRQEPNQGV 540

************************************************************

PAO1_1 QKQIDLFGAAERVGFYNTFAARALQDRIEIPEVGPIEISRDRETGEVHALRGPRFASMQF 600

PAO1_2 QKQIDLFGAAERVGFYNTFAARALQDRIEIPEVGPIEISRDRETGEVHALRGPRFASMQF 600

PA14_1 QKQIDLFGAAERVGFYNTFAARALQDRIEIPEVGPIEISRDRETGEVHALRGPRFASMQF 600

PA14_2 QKQIDLFGAAERVGFYNTFAARALQDRIEIPEVGPIEISRDRETGEVHALRGPRFASMQF 600

************************************************************

PAO1_1 HPESVLTREGPRIIADLLRHALVERRP 627

PAO1_2 HPESVLTREGPRIIADLLRHALVERRP 627

PA14_1 HPESVLTREGPRIIADLLRHALVERRP 627

PA14_2 HPESVLTREGPRIIADLLRHALVERRP 627

***************************

Alignment for PhzF:

CLUSTAL O(1.2.4) multiple sequence alignment

PAO1_1 MHRYVVIDAFASEPLQGNPVAVFFDCDDLSGERMQRMAREMNLSESTFVLRPQQDGDARI 60

PAO1_2 MHRYVVIDAFASEPLQGNPVAVFFDCDDLSGERMQRMAREMNLSESTFVLRPQQDGDARI 60

PA14_1 MHRYVVIDAFASEPLQGNPVAVFFDCDDLSGERMQRMAREMNLSESTFVLRPQQDGDARI 60

PA14_2 MHRYVVIDAFASEPLQGNPVAVFFDCDDLSGERMQRMAREMNLSESTFVLRPQQDGDARI 60

************************************************************

PAO1_1 RIFTPVNELPFAGHPLLGTAIALGAETDKDRLFLETRMGTVPFALERQDGKVVACSMQQP 120

PAO1_2 RIFTPVNELPFAGHPLLGTAIALGAETDKDRLFLETRMGTVPFALERQDGKVVACSMQQP 120

PA14_1 RIFTPVNELPFAGHPLLGTAIALGAETDKDRLFLETRMGTVPFALERQDGKVVACSMQQP 120

PA14_2 RIFTPVNELPFAGHPLLGTAIALGAETDKDRLFLETRMGTVPFALERQDGKVVACSMQQP 120

************************************************************

PAO1_1 IPTWEHFSRPAELLAALGLKGSTFPIEVYRNGPRHVFVGLESVAALSALHPDHRALCDFP 180

PAO1_2 IPTWEHFSRPAELLAALGLKGSTFPIEVYRNGPRHVFVGLESVAALSALHPDHRALCDFP 180

PA14_1 IPTWEHFSRPAELLAALGLKGSTFPIEVYRNGPRHVFVGLESVAALSALHPDHRALCDFP 180

PA14_2 IPTWEHFSRPAELLAALGLKGSTFPIEVYRNGPRHVFVGLESVAALSALHPDHRALCDFP 180

************************************************************

PAO1_1 DLAVNCFAGAGRHWRSRMFSPAYGVVEDAATGSAAGPLAIHLARHRQIPYGQQIEILQGV 240

PAO1_2 DLAVNCFAGAGRHWRSRMFSPAYGVVEDAATGSAAGPLAIHLARHRQIPYGQQIEILQGV 240

PA14_1 DLAVNCFAGAGRHWRSRMFSPAYGVVEDAATGSAAGPLAIHLARHRQIPYGQQIEILQGV 240

PA14_2 DLAVNCFAGAGRHWRSRMFSPAYGVVEDAATGSAAGPLAIHLARHRQIPYGQQIEILQGV 240

************************************************************

PAO1_1 EIGRPSRMYARAEGAGERVSAVEVSGNGAAFAEGRAYL 278

PAO1_2 EIGRPSRMYARAEGAGERVSAVEVSGNGAAFAEGRAYL 278

PA14_1 EIGRPSRMYARAEGAGERVSAVEVSGNGAAFAEGRAYL 278

PA14_2 EIGRPSRMYARAEGAGERVSAVEVSGNGAAFAEGRAYL 278

**************************************

Alignment for PhzG:

CLUSTAL O(1.2.4) multiple sequence alignment

PAO1_1 -MGVNANISESLTGTIEAPFPEFEAPPANPMEVLRNWLERARRYGVREPRALALATVDGQ 59

PAO1_2 MMGVNANISESLTGTIEAPFPEFEAPPANPMEVLRNWLERARRYGVREPRALALATVDGQ 60

PA14_1 MMGVNANISESLTGTIEAPFPEFEAPPANPMEVLRNWLERARRYGVREPRALALATVDGQ 60

PA14_2 MMGVNANISESLTGTIEAPFPEFEAPPANPMEVLRNWLERARRYGVREPRALALATVDGQ 60

***********************************************************

PAO1_1 GRPSTRIVVIAELGERGVVFATHADSQKGRELAQNPWASGVLYWRESSQQIILNGRAERL 119

PAO1_2 GRPSTRIVVIAELGERGVVFATHADSQKGRELAQNPWASGVLYWRESSQQIILNGRAERL 120

PA14_1 GRPSTRIVVIAELGERGVVFATHADSQKGRELAQNPWASGVLYWRESSQQIILNGRAERL 120

PA14_2 GRPSTRIVVIAELGERGVVFATHADSQKGRELAQNPWASGVLYWRESSQQIILNGRAERL 120

************************************************************

PAO1_1 PDERADAQWLSRPYQTHPMSIASRQSETLADIHALRAEARRLAETDGPLPRPPGYCLFEL 179

PAO1_2 PDERADAQWLSRPYQTHPMSIASRQSETLADIHALRAEARRLAETDGPLPRPPGYCLFEL 180

PA14_1 PDERADAQWLSRPYQTHPMSIASRQSETLADIHALRAEARRLAETDGPLPRPPGYCLFEL 180

PA14_2 PDERADAQWLSRPYQTHPMSIASRQSETLADIHALRAAARRLAETDGPLPRPPGYCLFEL 180

************************************* **********************

PAO1_1 CLESVEFWGNGTERLHERLRYDRDEGGWKHRYLQP 214

PAO1_2 CLESVEFWGNGTERLHERLRYDRDEGGWKHRYLQP 215

PA14_1 CLESVEFWGNGTERLHERLRYDRDEGGWKHRYLQP 215

PA14_2 CLESVEFWGNGTERLHERLRYDRDEGGWKHRYLQP 215

***********************************

**Supplementary Data 2 - Other regulatory elements**

Other intergenic regions presenting RBS have been detected but with no differences among them.

This region is located between phzE and phzF.

PAO1_1 gccaggagagccc

PAO1_2 gccaggagagccc

PA14_1 gccaggagagccc

PA14_2 gccaggagagccc

*************

This region is located between phzF and phzG.

PAO1_1 acgggcagaacggaggaacgcc

PAO1_2 acgggcagaacggaggaacgcc

PA14_1 acgggcagaacggaggaacgcc

PA14_2 acgggcagaacggaggaacgcc

**********************
